# Comparison of knowledge, attitude, practice and predictors of self-medication with antibiotics among medical and non-medical students in Tanzania

**DOI:** 10.3389/fphar.2023.1301561

**Published:** 2024-01-11

**Authors:** Lusajo Shitindi, Omary Issa, Baraka P. Poyongo, Pius Gerald Horumpende, Godeliver A. Kagashe, Raphael Z. Sangeda

**Affiliations:** ^1^ Department of Pharmaceutical Microbiology, Muhimbili University of Health and Allied Sciences, Dar es Salaam, Tanzania; ^2^ Department of Pharmaceutics and Pharmacy Practice, Muhimbili University of Health and Allied Sciences, Dar es Salaam, Tanzania; ^3^ Department of Biochemistry and Molecular Biology, Kilimanjaro Christian Medical University College, Moshi, Tanzania; ^4^ Kilimanjaro Clinical Research Institute (KCRI), Moshi, Tanzania; ^5^ Lugalo Infectious Diseases Hospital and Research Centre, General Military Hospital (GMH) and Military College of Medical Sciences (MCMS), Dar es Salaam, Tanzania

**Keywords:** self medication, antibiotic, adverse drug event, university student, medical students, Tanzania

## Abstract

**Introduction:** Self-medication with antibiotics (SMA) is a widespread problem in developing nations, including Tanzania.

**Methods:** This study compared knowledge, attitudes, practices, and factors influencing antibiotic SMA among medical and non-medical students.

**Results:** The prevalence of SMA among medical students was 49.1% and 59.2% among non-medical students, respectively. The mean knowledge score of medical students (6.4) was significantly higher (*p*-value <0.001) than that of non-medical students (5.6). The main factors influencing SMA practices were the availability of antibiotics without a prescription, easy access to pharmacies, and a lack of knowledge about the risks of SMA. This experience was pivotal in influencing medical students to take antibiotics, with a substantial proportion of 67.5% as opposed to 59.4% of non-medical students. Medical students were 1.6 times more likely to self-medicate with antibiotics than non-medical students (Adjusted Odds Ratio (AOR): 1.6; 95% Confidence Interval (CI): 1.2–2.3, *p*-value = 0.004). Age was also associated with self-medication, with an AOR of 1.1 (95% CI: 1.04–1.2, *p*-value = 0.006) per year increase in age. Additionally, attitude was associated with self-medication, with an AOR of 1.05 (95% CI: 1.04–1.1, *p*-value = 0.001) per unit increase in attitude score.

**Discussion:** No significant associations were found between sex, marital status, having children, year of study, knowledge score, and self-medication with antibiotics. This study emphasizes the importance of educational interventions and public awareness campaigns to promote antimicrobial stewardship, appropriate antibiotic use, and preventing pharmacies from dispensing antibiotics without a prescription.

## 1 Introduction

Self-medication with antibiotics (SMA) has been linked to an increase in antimicrobial resistance (AMR) (Konozy et al., 2015). AMR is a global health crisis that requires the involvement of all sectors. Antibiotics are frequently inappropriately used for specific ailments because of a lack of knowledge. Individuals and communities are at risk owing to various side effects, including antibiotic resistance (Shah et al., 2014). Therefore, the prevalence of SMA in developing countries must be investigated (Shah et al., 2014).

SMA is more prevalent in low—and middle-income countries (LMICs) than in high-income countries (HICs) (Pan et al., 2012). This is because of HIC’s strict antimicrobial use (AMU) regulatory requirements (Harbarth and Samore, 2005). LMICs such as Tanzania are particularly vulnerable to AMR because of their large populations and rising international travel rates. SMA is estimated to affect 3%–19% of the general population in developed countries and 9%–100% in developing countries, with consequences such as masking symptoms, treatment failure, drug resistance, and adverse drug events (ADEs), including death (Pan et al., 2012; Zhu et al., 2016).

According to global data, sub-Saharan Africa, including Tanzania, has the highest rate of infectious pathogens and AMR mortality (Ikuta et al., 2022; Murray et al., 2022). SMA is another significant contributor to antibiotic misuse, particularly given the availability of antibiotics from pharmacies in many developing countries (Banerjee et al., 2016). According to restrictions imposed by the China Food and Drug Administration in 2004, pharmacies can only sell prescribed antibiotics. Despite this, antibiotics are frequently obtained from pharmacies without a prescription (Chang et al., 2017; Mate et al., 2019; Poyongo and Sangeda, 2020).

A preliminary assessment revealed that the infrastructure for implementing AMR stewardship was successful, indicating a positive shift in practice (Sangeda et al.; Frumence et al., 2021). Tanzania began to increase AMR stewardship on a small scale in 2017 (Sangeda et al., 2020; Frumence et al., 2021). Evidence shows that medication from a qualified medical practitioner can be obtained without a prescription at a pharmacy in Tanzania (Mate et al., 2019; Poyongo and Sangeda, 2020), allowing people to practice SMA.

Several studies have described antibiotic-related behaviors among medical students in China, Ethiopia, India, Nigeria, Nepal, Pakistan, Rwanda, Europe, and the United States. The SMA ranges from 19% to 100% (Haltiwanger et al., 2001; Pulcini et al., 2015; Scaioli et al., 2015; Ali et al., 2016; Banerjee et al., 2016; Fejza et al., 2016; Zhu et al., 2016; Hu et al., 2018; Tuyishimire et al., 2019; Tesfaye et al., 2020; Akande-Sholabi and Ajamu, 2021; Nabi et al., 2022; Siraj et al., 2022).

Many studies have reported SMA frequencies, poor SMA practices such as frequent dosage or antibiotic changes, and SMA risk factors primarily related to sex, age, education level, antibiotic knowledge, and income; however, these risk factors vary across populations and countries and are thus controversial, and SMA practices of risk populations are rare (Haltiwanger et al., 2001; Pan et al., 2012; Pulcini et al., 2015; Scaioli et al., 2015; Ali et al., 2016; Banerjee et al., 2016; Fejza et al., 2016; Zhu et al., 2016; Hu et al., 2018; Tuyishimire et al., 2019; Tesfaye et al., 2020; Akande-Sholabi and Ajamu, 2021; Hsu, 2021; Nabi et al., 2022; Siraj et al., 2022).

According to previous studies, college students have a very low proclivity to seek health professionals’ health-related information, treatment, or other healthcare services (Hsu, 2021). Owing to increased social media influence, students have relied on the Internet for health information rather than consulting healthcare providers in recent years (Haltiwanger et al., 2001; Pulcini et al., 2015; Scaioli et al., 2015; Ali et al., 2016; Banerjee et al., 2016; Fejza et al., 2016; Zhu et al., 2016; Hu et al., 2018; Tuyishimire et al., 2019; Tesfaye et al., 2020; Akande-Sholabi and Ajamu, 2021; Nabi et al., 2022; Siraj et al., 2022). This increases the likelihood of college students using SMA to treat self-diagnosed illnesses (Tesfaye et al., 2020).

Doctors’ knowledge and behavior may cause high rates of antibiotic misuse and patient pressure (Hu et al., 2018). Previous routine clinical practice standards may have influenced public perception and led to an erroneous understanding of appropriate antibiotic use (Ali et al., 2016).

Medical students are especially important because they will be future clinic leaders in charge of antibiotic prescription and communication with their patients (Shah et al., 2014). Previous research has found that medical knowledge may influence college students’ SMA practices (Tesfaye et al., 2020). According to a study on AMR and its use in East Africa, final-year students have little knowledge of antimicrobial resistance and antibiotic use in clinical scenarios (Lubwama et al., 2021).

Only a few recent studies on medical students’ antibiotic use behavior have been conducted in Tanzania (Chuwa et al., 2021; Mabilika et al., 2022). Other general population studies have also been conducted (Horumpende et al., 2018; Marwa et al., 2018). It is unclear how much pre-medical education influences medical students’ KAP differently from non-medical students. As a result, four universities in Dar es Salaam, Tanzania, collaborated in this descriptive cross-sectional study to investigate the KAP and determinants of SMA.

## 2 Methodology

### 2.1 Study design and setting

This descriptive cross-sectional study was conducted at two medical and two non-medical universities in Dar es Salaam, Tanzania.

### 2.2 Study participants

The study was conducted at four universities (two medical and two non-medical) in Dar es Salaam, Tanzania. Dar es Salaam is the largest commercial city in Tanzania and serves as a university education hub.

All undergraduate students from the four universities from the first to the fifth year were enrolled regardless of the program.

### 2.3 Study area

The study included medical students from the Muhimbili University of Health and Allied Sciences (MUHAS) and Kampala International University in Tanzania (KIUT). MUHAS and KIUT are two public and private academic institutions in Dar es Salaam’s Ilala district, respectively, which are among Tanzania’s medical universities. The non-medical students surveyed were from the University of Dar-es-Salaam (UDSM) and Ardhi University (ARU) in the Ubungo District, Dar es Salaam region.

The MUHAS is located in the Ilala district. Currently, it has a carrying capacity of approximately 3,500 students. Students are educated in five schools at the university: the School of Medicine, the School of Pharmacy, the School of Dentistry, the School of Nursing, and the School of Public Health, to which all students are admitted. Both undergraduate and postgraduate students were given medical training at the university. Undergraduate students are taught various programs such as MD, pharmacy, nursing, laboratory science, radiotherapy, environmental health science, and DDS in different years.

The Julius Nyerere International Airport in Gongo la Mboto is 5 km from KIUT. The university offers three undergraduate medical courses: a bachelor’s degree in medicine and surgery, medical laboratory sciences, pharmacy, and non-medical.

### 2.4 Sample size calculation

The required sample size was determined using the formula N = Z^2^P(1 - P)/E^2^ (Naing et al., 2022). In this formula, “N” denotes the sample size, “Z” is the standard normal deviation—usually set at 1.96 to correspond to a 95% confidence interval, “P” is the assumed prevalence, and “E” stands for the acceptable margin of error. Given this study’s lack of known prevalence, we used a conservative estimate of 50% (*p*-value = 0.5) to maximize variability. The acceptable margin of error (E) was 5% (*p*-value = 0.05). After applying these values to the formula, we calculated the sample size of approximately 384 participants. Despite the estimated sample size 384, we ultimately included all the surveyed students from the four selected universities in our final analysis. The decision to exceed the initially calculated sample size was aimed at enhancing our study’s statistical power and ensuring the robustness of our findings. Notably, expanding the sample size in this manner did not contravene ethical guidelines nor significantly inflate the resource implications of the study.

### 2.5 Sampling technique

A convenience sampling technique was used to obtain a sample of students for this study. All non-medical undergraduate students at the University of Dar es Salaam and Ardhi University were invited to participate in this study. Participants were chosen from a predetermined pool of respondents. This technique was appropriate for this study because it was simple, used participants found during data collection time, was cost-effective, and did not necessitate any follow-up.

### 2.6 Data collection and questionnaire

SMA was defined as self - treatment with non - prescription (over - the - counter) antibiotics was defined as self - treatment with non - prescription (over-the-counter) antibiotics. PKA was taught in medical schools through formal lectures on antibiotics.

A well-structured and pre-tested online questionnaire (Supplementary Material S1.1) with open and closed-ended questions was used to collect student data. The questionnaires were designed to cover the objectives and sociodemographic characteristics of the respondents in the four sections.

The first section discusses the sociodemographic characteristics of the respondents, such as their gender. The first section examined the respondents’ sociodemographic characteristics such as sex, age, university, faculty or school, year of study, residence, marital status, and parental status. The second section covered the practice of SM, including the frequency of SMA practice, commonly used antibiotics, symptoms that were mainly experienced, places where they were obtained, and the reasons that made them practice SMA. The third section tested the medical students’ understanding of antibiotics and SMA. The fourth section assessed the medical students’ attitudes towards SMA using a five - point Likert scale (strongly disagree to agree strongly). Respondents were asked to complete the questionnaires independently, following the instructions provided by the link provided. Responses were scored from 1 (strongly disagree) to 5 (strongly agree).

Responses are ranked from 1 (strongly disagree) to 5 (strongly agree). The specific-necessity and specific-concerns measures comprised five items, with values ranging from 5 to 25. The score was calculated by adding the points on each scale. Higher scores indicated a stronger belief in the concepts represented by the scale. Data were gathered using self-administered semi-structured questionnaires. Data were collected using self -\ self-administered semi-structured questionnaires.

### 2.7 Questionnaire

Data were gathered using self-administered semi-structured questionnaires. Students collected online data from standardized online questionnaires using REDCap software after the introduction text was presented (Supplementary Material S1.1).

Four KAP constructs were included in the questionnaire regarding antibiotic use for self-limiting illnesses and syndromes, such as the common cold, fever, sore throat, headache, ear pain, diarrhea, and abdominal pain. Students were asked to identify the antibiotics they had used and whether they were chemical or brand-named. Responses were yes/no or unknown/uncertain for each item. The behavioral outcomes of interest were SMA in the previous month for self-limiting illness, demanding an antibiotic from a clinician, stocking an antibiotic supply at home, and using antibiotics to prevent the common cold in the previous year. A detailed questionnaire is provided in the Appendix in Supplementary Materials S1.2, S1.3.

The first section covered the sociodemographic characteristics of the respondents, such as sex, age, university, faculty or school, year of study, residence, marital status, and parental status. The second section covered the practice of SM, including the frequency of practice of SMA, commonly used antibiotics, symptoms that were mainly experienced, places where they were obtained, and the reasons for the practice of SM. The third section focuses on medical students’ antibiotic knowledge and SMA. The fourth section covered the attitudes of medical students towards SMA. Participants’ responses were evaluated using a five-point Likert scale (strongly disagree - strongly agree). The respondents’ questionnaires were completed independently, following the instructions in each section. The REDCap website was used to invite students to participate anonymously in the study. Class representatives distributed the survey to students during free periods to encourage participation. Screening the IP address, submission time, and answering patterns prevented the same student involvement. The analysis only included completed questionnaires submitted by the students.

### 2.8 Pilot study

Before conducting the full study, 20 students participated in the trial studies, and the pilot study respondents were from various courses. This allowed us to test the study tools and approaches for translating the study’s theoretical picture into practice. This also helped to reveal the challenges that the study faced in increasing preparedness. The methodology, particularly the study tools, was modified to fit the environment demonstrated by the pilot study for efficient and effective data collection, thereby increasing its usefulness. Pilot-testing data were not used in the final analysis.

### 2.9 Attitude of students on self-medication with antibiotics

Attitude was scored by adding questions on various aspects of the SMA. Similarly, all practice questions were added to gauge a score for the standards of practice towards SMA.

Ten questions were asked to assess students’ attitudes towards SMA. Each positive attitude per question was scored using Likert points, and each student’s overall score was calculated.

### 2.10 Students’ level of knowledge on self-medication with antibiotics

Nine questions were asked to assess students’ understanding of SMA and the effects of SMA practice on patients and the community. Each correct response was awarded ten marks and at the end, the overall score was calculated for each student. A Likert scale was used to categorize students depending on their overall score.

### 2.11 Ethical considerations

Ethical approval to conduct the study was obtained from the MUHAS Senate Research and Publication Committee, with ethical clearance reference DA.25/111/01/03/2019. Consent was obtained from all participants before they were included in the study. The participants were guaranteed anonymity and confidentiality during the data presentation and publication.

### 2.12 Statistical data analysis

Cleaned data were analyzed using SPSS version 26.0. The data were extracted from the REDCap software in the SPSS format (.sav file). Incomplete responses were detected and excluded from the final dataset.

The Chi-square test and unconditional logistic regression were used to assess the relationships between antibiotic knowledge, attitudes, and SMA practices/attitudes of self-medicated students. The chi-square test was used to examine the association between demographic variables (sex, age, major, education level, category of education, type of hostel, and family status) and SMA. Variables with significant differences were included in bivariate correlation and unconditional logistic regression analyses to identify SMA risk factors. The data reliability for the ten attitude questions was tested using Cronbach’s alpha coefficient.

The data were entered into a REDCap database and analyzed using the SPSS statistical package version 26. The analysis included individuals who consented to answer all the questions. The results of the descriptive analysis are presented in tables and graphs. Fisher’s and Chi-squared tests were used for categorical variables, and the Wilcoxon signed-rank test for continuous values was used to test for association. Statistical significance was set at *p*-value <0.05. A logistic regression model determined the association between independent sociodemographic, knowledge and attitude characteristics and dependent SMA outcome. Variables that were significant in multivariate analysis was performed on variables that were significant in univariate analysis. (OR) and 95% confidence interval (CI).

## 3 Results

### 3.1 Social demographics information

A total of 929 students were invited to participate in the study, with one declining student and 99 others providing incomplete responses, yielding an analysis of 829 participants. There were 48.2% (400) and 429 medical students (51.8%) non-medical students among the participants.

In total, 36.4% of the respondents were female and 63.6% were male. MUHAS had the highest proportion of medical students who participated in the study (68.3%), while UDSM had the highest proportion of non-medical students (65.3%). Overall, 64.3% (533) of the respondents stayed in hostels. However, non-medical students (71.6%) were significantly more likely to reside in hostels than medical students (56.5%) (*p*-value <0.001) (Table 1).

**TABLE 1:** Social-demographic characteristics of medical and non-medical students.

Variable	Response	Medical student [N (%)]	Non-medical student [N (%)]	Total	*p*-value
Name of the university	MUHAS	273 (68.3%)	0 (0.0%)	273 (32.9%)	**<0.001**
	KIUT	127 (31.8%)	0 (0.0%)	127 (15.3%)	
	UDSM	0 (0.0%)	280 (65.3%)	280 (33.8%)	
	ARU	0 (0.0%)	149 (34.7%)	149 (18.0%)	
Year of study	First-year	57 (14.2%)	83 (19.3%)	140 (16.9%)	**<0.001**
	Second year	48 (12.0%)	171 (39.9%)	219 (26.4%)	
	Third year	132 (33.0%)	145 (33.8%)	277 (33.4%)	
	Fourth-year	163 (40.8%)	30 (7.0%)	193 (23.3%)	
Gender	Female	140 (35.0%)	162 (37.8%)	302 (36.4%)	0.409
	Male	260 (65.0%)	267 (62.2%)	527 (63.6%)	
Marital status	Single	374 (93.5%)	415 (96.7%)	789 (95.2%)	0.097
	Married	23 (5.8%)	14 (3.3%)	37 (4.5%)	
	Divorced	2 (0.5%)	0 (0.0%)	2 (0.2%)	
	Widower	1 (0.3%)	0 (0.0%)	1 (0.1%)	
Place of residence	Hostels	226 (56.5%)	307 (71.6%)	533 (64.3%)	<0.001
	Home	174 (43.5%)	122 (28.4%)	296 (35.7%)	
Have you ever heard of the term self-medication?	No	22 (5.5%)	115 (26.8%)	137 (16.5%)	<0.001
	Yes	378 (94.5%)	314 (73.2%)	692 (83.5%)	
Do you have child(ren)?	No	368 (92.0%)	403 (93.9%)	771 (93.0%)	0.274
	Yes	32 (8.0%)	26 (6.1%)	58 (7.0%)	
Number of children group (N = 33)	1	23 (69.7%)	22 (84.6%)	45 (76.3%)	0.364
	2	4 (12.1%)	1 (3.8%)	5 (8.5%)	
	>2	6 (18.2%)	3 (11.5%)	9 (15.3%)	
	Total	33 (100.0%)	26 (100.0%)	59 (100.0%)	
Have you ever been sick and obtained the medication yourself before visiting the doctor/physician in a hospital or clinic (self-medication)?	No	31 (7.8%)	36 (8.4%)	67 (8.1%)	0.7348
	Yes	369 (92.3%)	393 (91.6%)	762 (91.9%)	
Self-medicated antibiotics	No	203 (50.7%)	175 (40.8%)	378 (45.6%)	0.004
	Yes	197 (49.3%)	254 (59.2%)	451 (54.4%)	
Age group	18–23	186 (46.5%)	344 (80.2%)	530 (63.9%)	<0.001
	24–29	195 (48.8%)	80 (18.6%)	275 (33.2%)	
	≥30	19 (4.8%)	5 (1.2%)	24 (2.9%)	
Age	Mean	24	22.5		<0.001
	stdev	2.8	1.9		
Knowledge Score Mean (standard deviation)	Mean	6.4	5.6		<0.001
	stdev	1.1	1.18		
Attitude Score Mean (standard deviation)	Mean	23.0	28.0		<0.001
	stdev	6.0	4.3		
Total		400 (100%)	429 (100%)	829 (100%)	

Key: MUHAS, Muhimbili University of Health and Allied Sciences; KIUT, Kampala International University in Tanzania; UDSM, University of Dar - es - Salaam; ARU, Ardhi University (ARU). Significant *p*-values are shown in bold face.

Medical students were overrepresented (40.8%) in the fourth year, whereas non-medical students were overrepresented (39.9%) in the second year (*p*-value <0.001).

The year of study, age, attitude score, and knowledge score significantly differed between the medical and non-medical students. Medical students were more familiar with the term “self-medication” than non-medical students.

Non-medical students were older (80.2%) than medical students (48.8%) (*p*-value <0.001). Medical students, On average, were slightly older than non-medical students.

Medical students outperformed non-medical students in terms of mean knowledge, whereas non-medical students outperformed medical students in terms of mean attitude. Medical students outperformed non-medical students in terms of mean knowledge, whereas non-medical students outperformed medical students in terms of mean attitude.

There was no statistically significant difference in the sex distribution of medical and non-medical students. Most participants were single, and there was no statistically significant difference between medical (93.5%) and non-medical (96.7%) students in the marital status distribution. There was also no significant difference in the distribution of children between the medical and non-medical students (Table 1). There was also no significant difference in the distribution of children between the medical and non-medical students (Table 1).

### 3.2 Self-medication practice with any medicine or self-medication with antibiotics among students from non-medical universities

When asked if they had ever practiced SM, most participants had no significant difference between medical (92.3%) and non-medical (91.6%) students. However, non-medical students (59.2%) self-medicated with antibiotics (SMA) more frequently than medical students (49.3%) (Table 1).

### 3.3 Groups of medications used by students for self-medication

Of the interviewed students, 370 (92.3%) medical students and 393 (91.6%) non-medical students admitted to having ever self - medicated and only 197 (49.1%) and 254 (59.2%) medical and non-medical students were self-medicated with antibiotics, respectively, revealing that more non-medical students self - medicated with antibiotics than medical students (*p*-value = 0.004). However, the trend of SM with painkillers was the opposite, with more medical students, 333 (83.0%), admitting to self-medication with painkillers than non-medical students, 284 (66.2%) (*p*-value <0.001). There were no observed practice differences between medical and non-medical students in SM with an antifungal (*p*-value = 0.646) and medicine for treating chronic diseases (*p*-value = 0.136).

**FIGURE 1:**
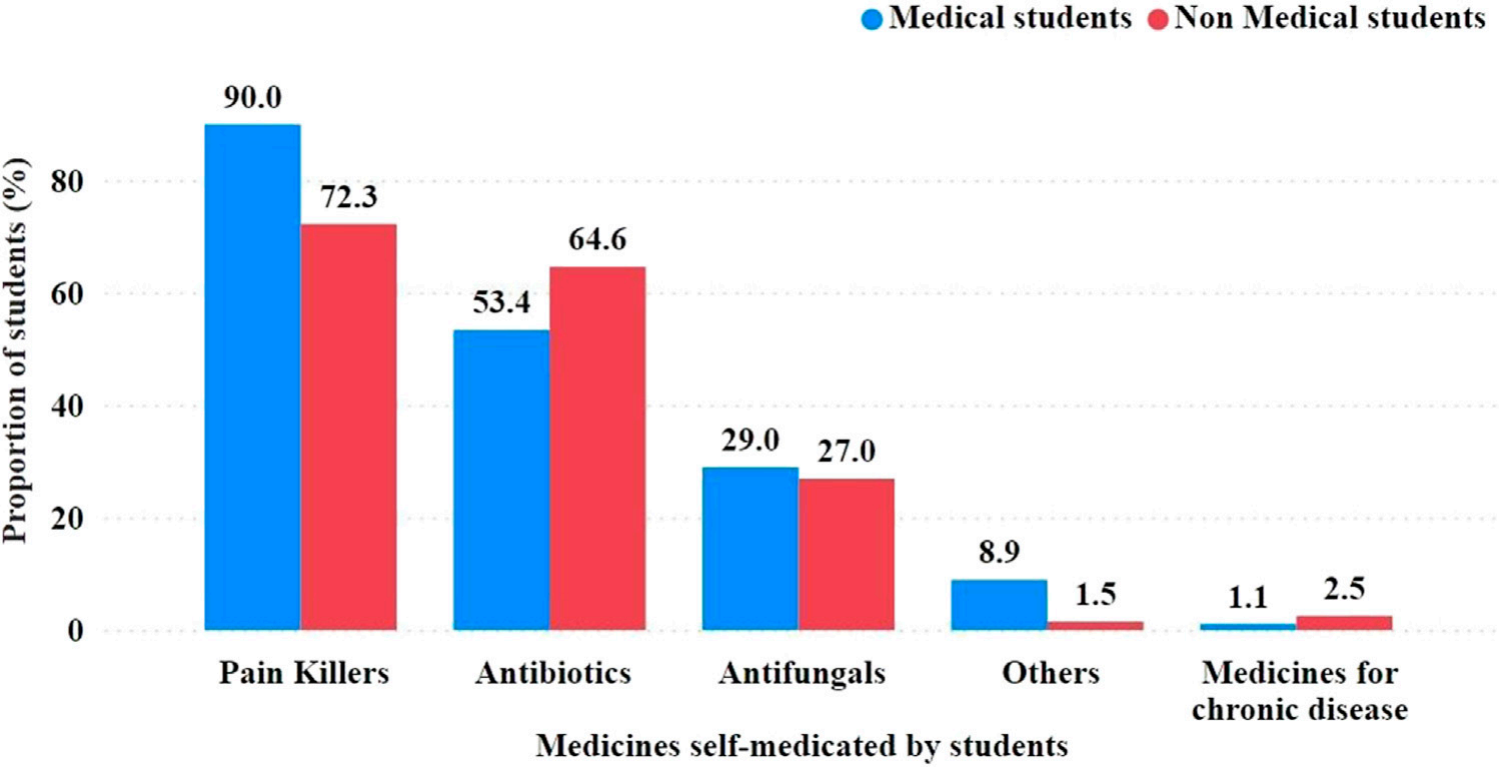
Groups of medications used by students for self-medication. Mentioned examples were included in the questionnaire to prompt the respondents on grouping medications and for easy recall. Exact antibiotics are required in Figure 4.

Types of medications investigated for SM by students. The most commonly used medications were pain relievers, such as paracetamol, ibuprofen, and meloxicam, reported by 90.0% of medical students and 72.3% of non-medical students (Figure 1).

Antibiotics were another commonly used type of medication, reported by 53.4% of medical students and 64.6% of non-medical students. Among the antibiotics used were penicillins, such as amoxicillin; cephalosporins, such as cephalexin; and tetracyclines, such as doxycycline and macrolides.

Antifungals, used to treat fungal infections, were used by a lower proportion of SMA students, with 29.0% of medical students and 27.0% of non-medical students reporting that they had used them. Miconazole, griseofulvin, fluconazole, and nystatin are examples of antifungal agents.

Use of medications for chronic diseases such as heart disease, hypertension, kidney disease, and diabetes. Only 1.1% of medical and 2.5% of non-medical students reported using these medications for SM.

Because a respondent could select more than one group of SM medications, the percentages for each group were not mutually exclusive.

### 3.4 Sources students hear about self-medication

**FIGURE 2:**
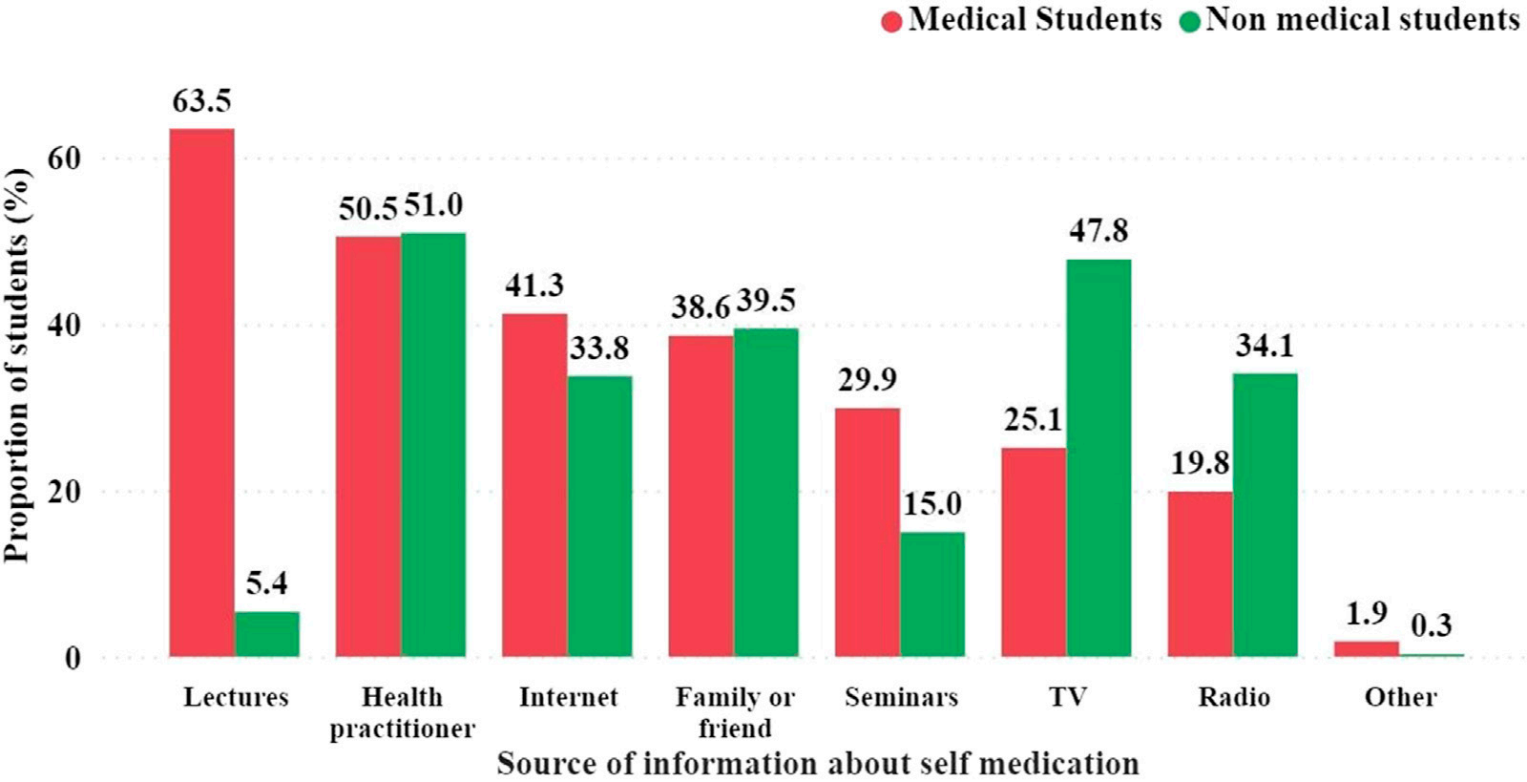
Sources students hear about self-medication.

Health practitioners frequently inform students about SM, with 50.5% of medical students and 51.0% of non-medical students citing it as a source. Furthermore, lecturers taught SM to a significantly higher proportion of medical students (63.5%) than non-medical students (5.5%) (Figure 2).

### 3.5 Sources influencing students to take antibiotics

**FIGURE 3:**
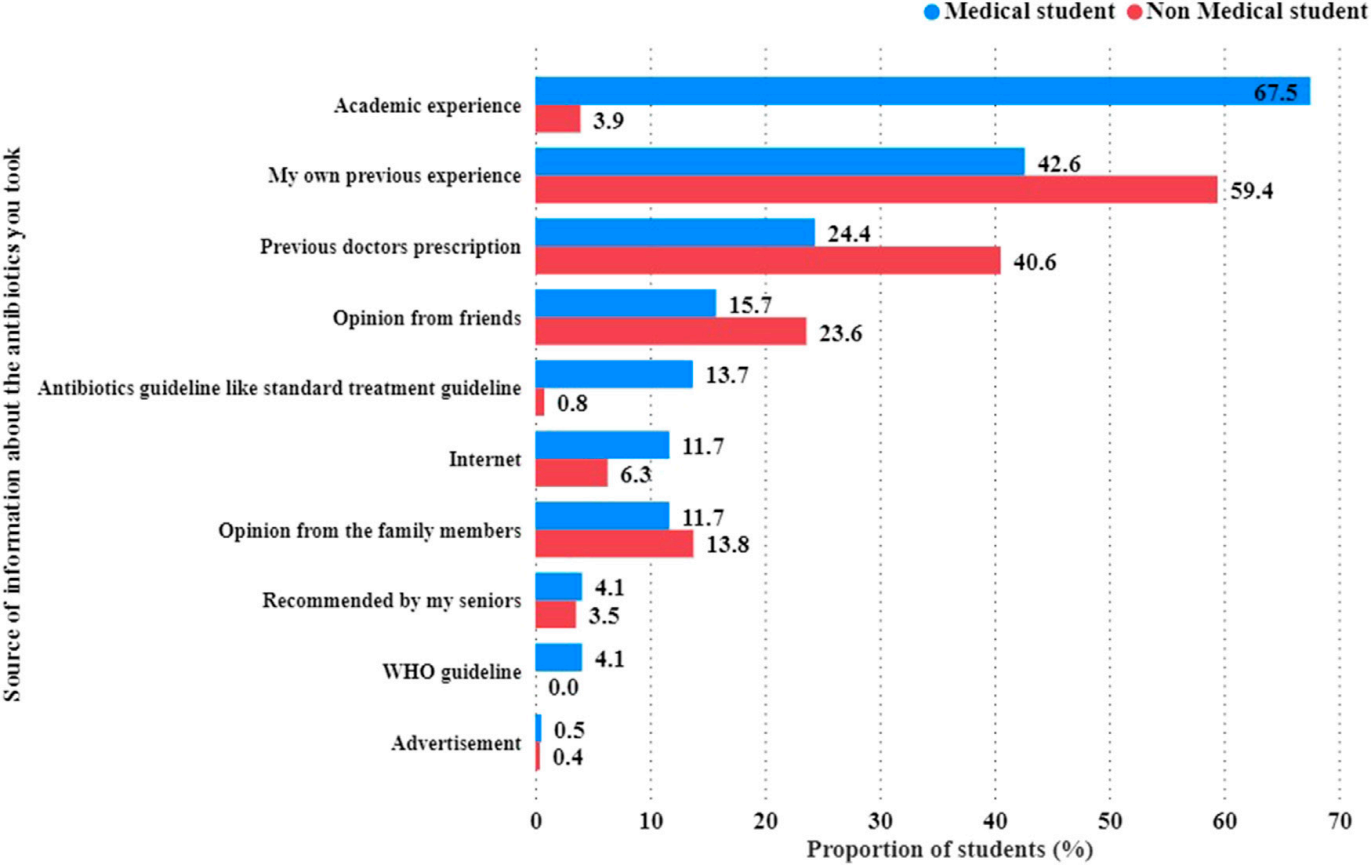
Sources influencing students to take antibiotics.

Academic experience was the most important factor driving medical students to take antibiotics, with a proportion of 67.5% compared to 3.9% of non-medical students. In contrast, 59.4% of non-medical students were more likely than 42.6% of medical students to base their decisions on prior experiences (Figure 3).

Aside from academic and personal experiences, other motivators for taking antibiotics included a previous doctor’s prescription, a friend’s opinion, standard antibiotic guidelines, Internet sources, family members’ opinions, senior recommendations, WHO guidelines, and advertisements.

Regarding the use of TV as a source of SM information, non-medical students (47.8%) outnumbered medical students (25.1%). A similar pattern was observed when radio was used as the source of SM information. Notably, the respondents were allowed to select more than one source of SM information (Figure 3).

### 3.6 Self-medication with antibiotics

When medical and non-medical students in Dar es Salaam, Tanzania, SM was compared with antibiotics. The term “self-medication” was heard by 83.6% of the 451 participants, with a significant difference between medical and non-medical students (95.4% vs. 74.4%, *p*-value = 0.001)). More than half of the participants (57.4%) had practiced SM in the past month, with significantly higher rates among non-medical students (70.1% vs. 41.1%, *p*-value <0.001). Most participants who reported self-medication did so 1–2 times a year (49.2%), with fewer reporting 3–5 times yearly (35.5%). Only 9.3% of the participants reported experiencing serious side effects or adverse drug reactions following SM with antibiotics, with a higher proportion among non-medical students (12.2% vs. 5.6%, *p*-value = 0.016). Among those who experienced side effects, the majority (69%), with a significantly higher proportion among non-medical students (80.6% vs. 36.4%, *p*-value = 0.012), continued to use medications (Table 2).

**TABLE 2:** Comparison of self-medication with antibiotics among medical and non-medical students in Dar es Salaam, Tanzania (N = 451).

Variable	Response	Medical student [N (%)]	Non-medical student [N (%)]	Total	Chi-square *p*-value
Have you ever heard of the term self-medication?	No	9 (4.6%)	65 (25.6%)	74 (16.4%)	**<0.001**
	Yes	188 (95.4%)	189 (74.4%)	377 (83.6%)	
When did you practice self-medication?	In the last 1 month	81 (41.1%)	178 (70.1%)	259 (57.4%)	**<0.001**
	In the last 6 months	76 (38.6%)	65 (25.6%)	141 (31.3%)	
	In the last 1 year	15 (7.6%)	9 (3.5%)	24 (5.3%)	
	More than 1 year ago	25 (12.7%)	2 (0.8%)	27 (6%)	
How many times did you practice self-medication in the period above?	1–2 times a year	97 (49.2%)	125 (49.2%)	222 (49.2%)	**0.003**
	3–5 times a year	61 (31%)	99 (39%)	160 (35.5%)	
	6–12 time	13 (6.6%)	20 (7.9%)	33 (7.3%)	
	more than 12 times	26 (13.2%)	10 (3.9%)	36 (8%)	
Total		197 (100%)	254 (100%)	451 (100%)	
Have you ever experienced severe side effects or any adverse drug reaction following self-medication with antibiotics	No	186 (94.4%)	223 (87.8%)	409 (90.7%)	**0.016**
	Yes	11 (5.6%)	31 (12.2%)	42 (9.3%)	
What did you do when encountering such side effects or adverse drug reactions? (N = 42)	I continued to use the medicines	4 (36.4%)	25 (80.6%)	29 (69%)	**0.012**
	I stopped taking the medicines immediately	5 (45.5%)	3 (9.7%)	8 (19%)	
	I stopped taking the medication immediately and visited the physician	2 (18.2%)	1 (3.2%)	3 (7.1%)	
	I have never experienced any side effects with the antibiotics I used	0 (0%)	2 (6.5%)	2 (4.8%)	
Total		197 (100%)	254 (100%)	451 (100%)	

Key: Significant *p*-values are shown in bold face.

### 3.7 Types of antibiotics and other antimicrobials used by students for self-medication N = 393

**FIGURE 4:**
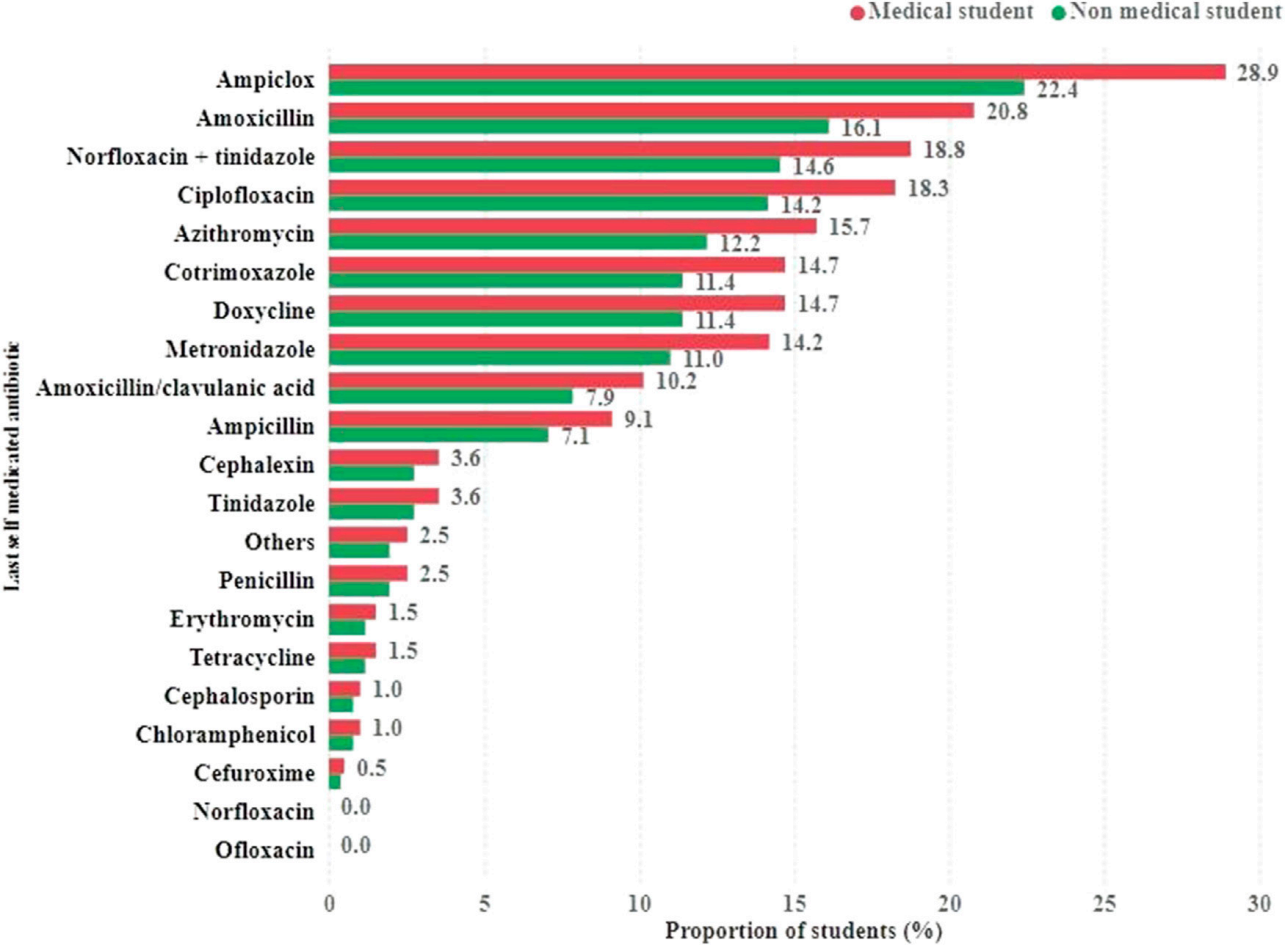
Types of antibiotics and other antimicrobials used by students for self-medication.

Based on this study, 393 students reported using antibiotics or other antimicrobials for SM. The most commonly used antibiotics were ampiclox (28.9% medical and 22.4% non-medical) and amoxicillin (20.8% medical and 16.1% non-medical). Other widely used antibiotics include norfloxacin, tinidazole, ciprofloxacin, azithromycin and cotrimoxazole. In each category, the proportion of medical students who self-medicated with the specified antibiotic was higher than that of the non-medical students. Notably, the respondents could choose more than one type of antibiotic for SM (Figure 4).

### 3.8 Symptoms or illness that made students practice self-medication with antibiotics N = 393

**FIGURE 5:**
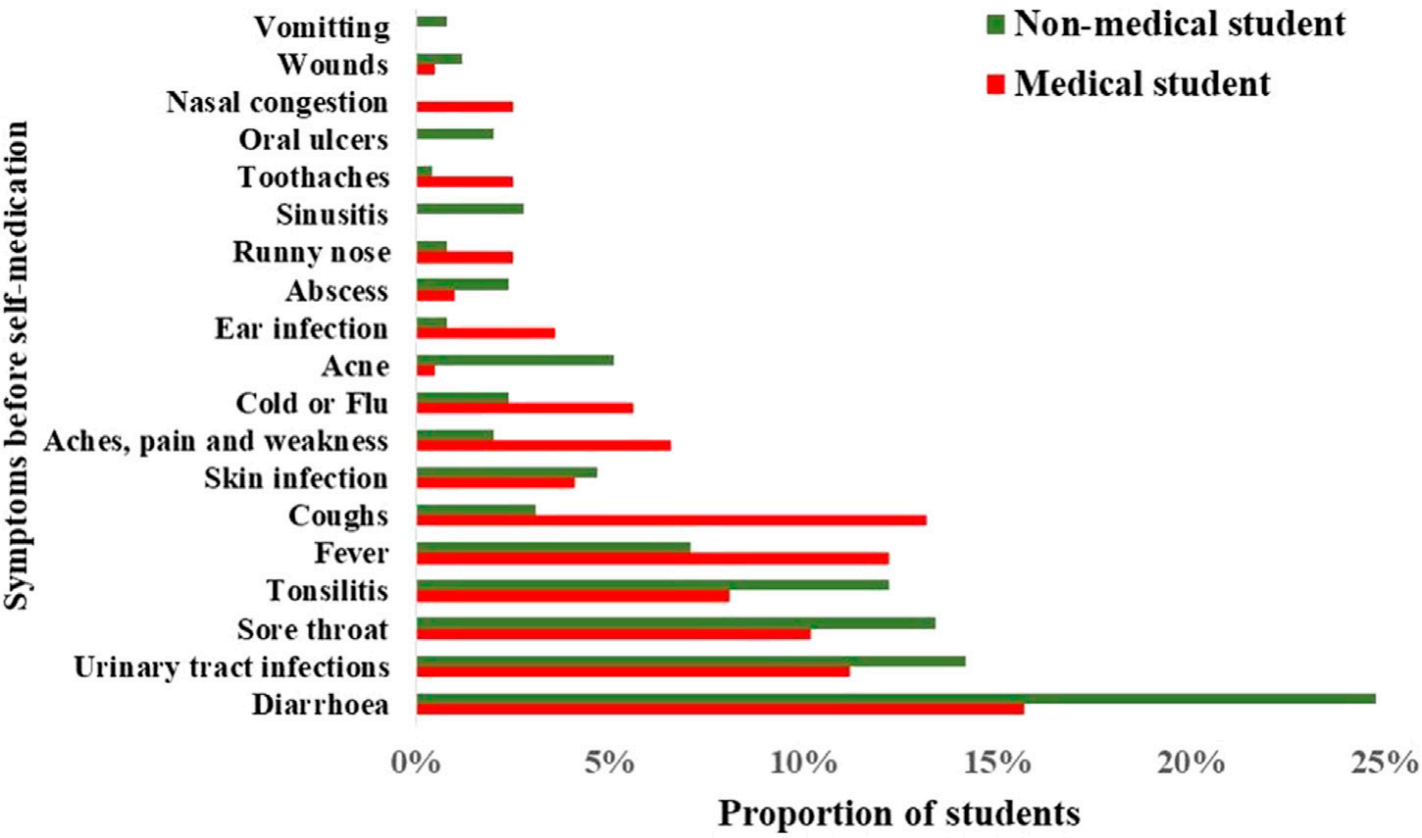
Symptoms or illnesses that led students to practice self-medication.

The table shows the symptoms and illnesses that led medical and non-medical students to use antibiotics. The most common reason for using antibiotics was diarrhea, which was reported by 20.8% of all respondents, with a higher prevalence among non-medical students (24.8%) than medical students (15.7%) (Figure 5). Other common reasons for antibiotic use included urinary tract infections (12.9%), sore throat (12.0%), and tonsillitis (10.4%). The less common reasons for using antibiotics were wounds (0.9%), oral ulcers (1.1%), and nasal congestion (1.1%). The prevalence of each symptom varied between the medical and non-medical students. A *p*-value of 0.000 suggested that the difference in symptom prevalence between the two groups was statistically significant.

### 3.9 Reasons for self-medication practice among the students N = 393

**FIGURE 6:**
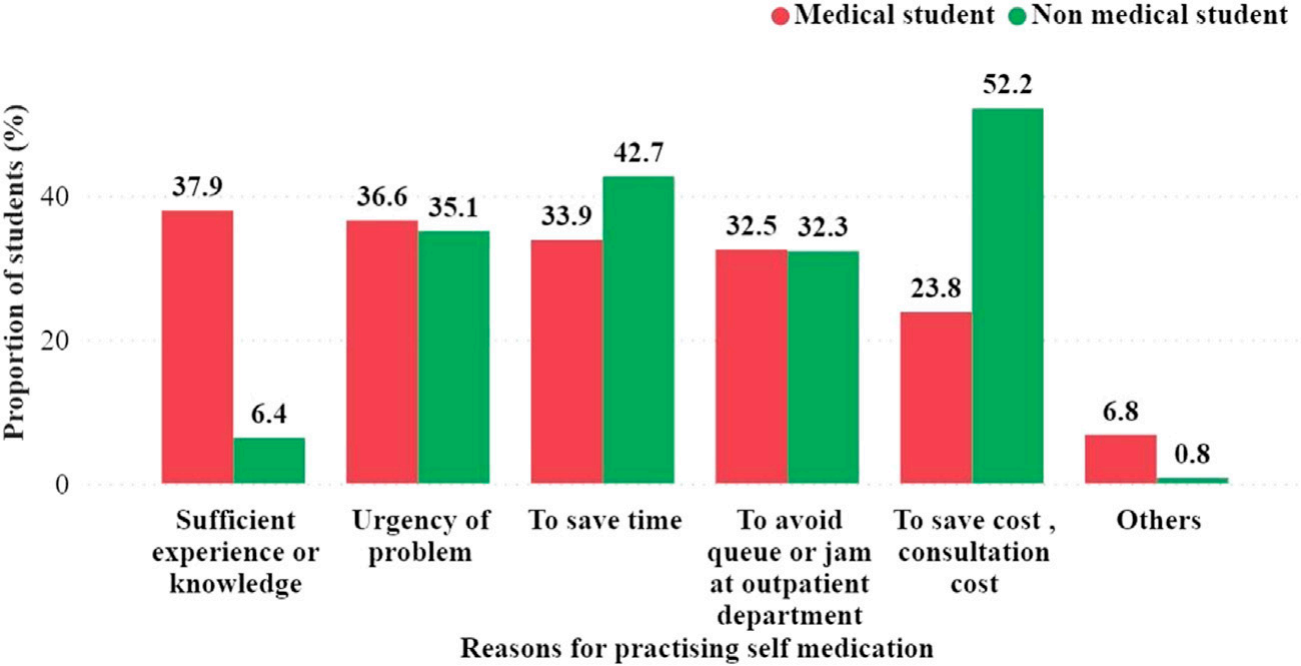
Types of antibiotics and other antimicrobials used by students for self-medication.

Among the students surveyed, it was found that medical and non-medical students self-medicated with antibiotics for various reasons. More medical students (37.9%) cited sufficient experience or knowledge as a reason for SM than non-medical students (6.4%). In contrast, a higher percentage of non-medical students (52.2%) reported practicing SM to save consultation costs than medical students (23.8%) (Figure 6). Other reasons for SM cited by both groups included the urgency of the problem and the avoidance of queues or jams in outpatient departments (Figure 6).

### 3.10 The sources of information on self-medication with antibiotics among the students N = 393

According to the study, 35.2% of students used their own previous experience, and 24% used a previous doctor’s prescription to seek medication. Other sources included 14% from friends, 8.2% from family, 3.7% from the Internet, 2.1% from elders, 0.5% from antibiotic patient leaflets, and 0.2% from advertisements.

### 3.11 Sources where students obtain antibiotics for self-medication N = 393

**FIGURE 7:**
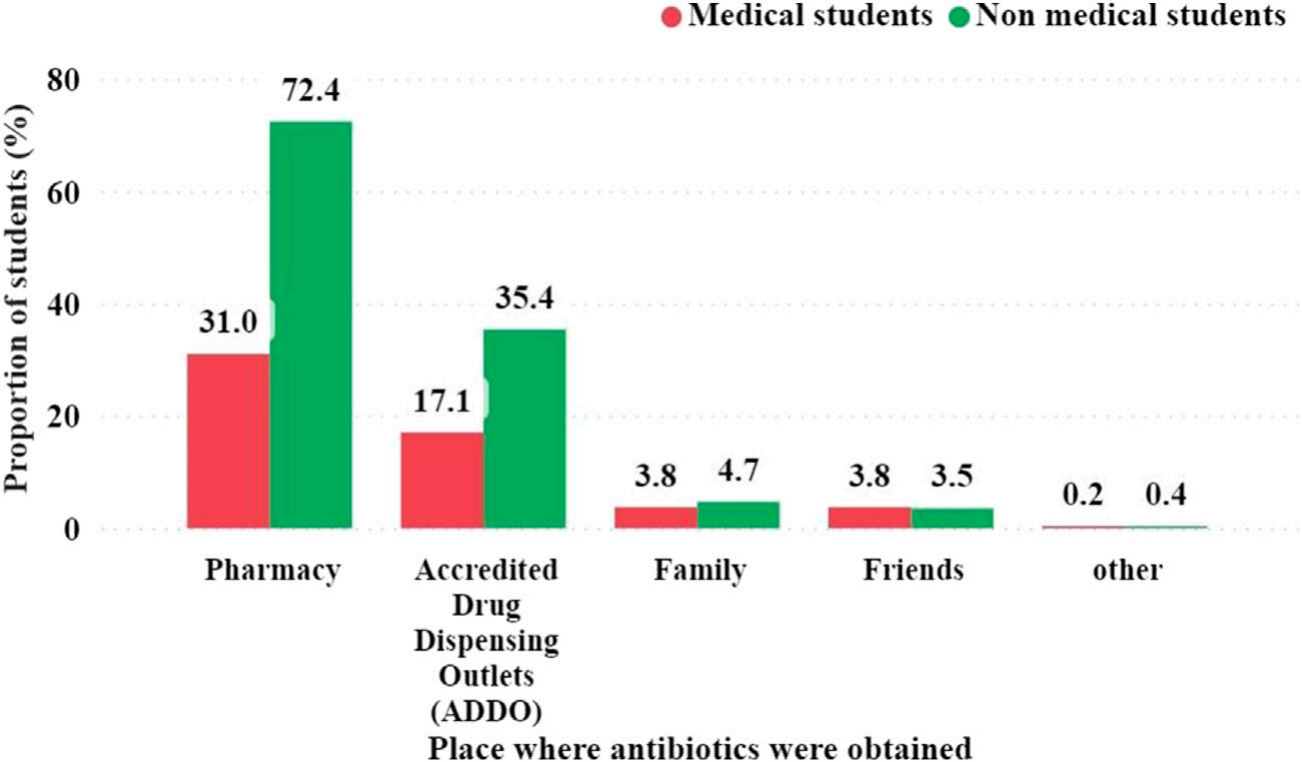
Places where students obtain antibiotics for self-medication N = 451.

Antibiotics were obtained from a variety of sources, including pharmacies, accredited drug dispensing outlets (ADDO), friends, and family members. SM medications were obtained from various sources, including pharmacies, accredited drug dispensing outlets (ADDO), friends, and family members. Self-medication products were obtained from various sources, including pharmacies, accredited drug dispensing outlets (ADDO), friends, and family members (Figure 7). Among the medical students, 31.0% obtained antibiotics from pharmacies, whereas 17.1% obtained them from ADDOs. In contrast, among non-medical students, 72.4% received antibiotics from pharmacies and 35.4% from ADDOs, locally known as “Duka la Dawa.”

### 3.12 Students’ level of knowledge on self-medication with antibiotics

This study also assessed the knowledge and attitudes of medical and non-medical students towards SM with antibiotics. The results showed that The mean knowledge score of medical students (6.4) was significantly higher (*p*-value <0.001) than that of non-medical students (5.6), indicating a better understanding of antibiotics and SM among medical students (Table 1).

### 3.13 Attitude of students on SM with antibiotics

Statistical techniques revealed significant associations supported by a scale with a Cronbach’s alpha coefficient of 0.691, indicating good internal consistency of the attitude scale.

The mean attitude score of non-medical students (28.0) was significantly higher (*p*-value <0.001) than that of medical students (23.0), suggesting a more positive attitude towards SM among non-medical students (Table 1).

### 3.14 Factors associated with self-medication with antibiotics (*n* = 892)

This study examined factors associated with SMA among 892 students. The adjusted odds ratio (AOR) for being a medical student was 1.6 (95% CI: 1.2–2.3, *p*-value = 0.004), indicating that medical students were more likely to self-medicate with antibiotics than non-medical students. Age was also associated with SM, with an AOR of 1.1 (95% CI: 1.03–1.2, *p*-value = 0.006) per year increase in age. Students’ attitude scores were also associated with SMA, with an AOR of 1.05 (95% CI: 1.02–1.1, *p*-value = 0.001) per unit increase in attitude scores. No significant association was found between sex, marital status, having children, year of study, knowledge score, and SMA (Table 3).

**TABLE 3:** Factors associated with self-medication with antibiotics (*n* = 892).

Variable	Attribute	cOR	*p*-value	AOR	*p*-value
Medical student	Yes	1.5 (1.14–2.0)	0.004	1.6 (1.2–2.3)	**0.004**
Gender	Female	1.1 (0.8–1.4)	0.739		
Marital status	Single	1.0	1.000		
	Married	1.0 (0.5–1.9)	0.970		
	Divorced	135.7 (0–0)	0.999		
	Widower	0 (0–0)	1.000		
Age		1.1 (1.03–1.2)	0.004	1.1 (1.04–1.2)	**0.006**
Year of study	First year	1	0.077	1	0.080
	Second year	1.6 (1.01–2.4)	0.044	1.2 (0.8–2.0)	0.389
	Third year	1.362 (0.9–2.0)	0.137	1.1 (0.7–1.7)	0.758
	Fourth-year	1.8 (1.1–2.7)	0.013	1.8 (1.1–3.0)	0.032
Do you have child(ren)	Yes	0.6 (0.4–1.1)	0.081	1.0 (0.5–2.0)	0.971
Attitude score		1.04 (1.01–1.1)	0.001	1.1 (1.02–1.2)	**0.001**
Knowledge score		1.0 (0.9–1.2)	0.485		

OR, odds ratio; aOR, adjusted odds ratio; CI, confidence interval.

Key: Significant *p*-values are shown in bold face.

## 4 Discussion

This study aimed to assess the knowledge, attitudes, and practice of SMA among medical students in Tanzania. Studies on SMA are essential to examine the extent of the problem and hence advice on the prevention of the consequences associated with SMA, as medical students play a significant role in healthcare care decision-making, and they represent a major part of a community that is highly qualified, trained, and superior in medical and health-related information.

SMA prevalence varies across countries and regions. In this study, the rate of SMA practice was 49.1%, which is consistent with a study in South India (48%) (Kumar et al., 2013) and Iran (Sarahroodi et al., 2009), but higher than that of Nigeria (Ehigiator et al., 2013), Uganda (Niwandinda et al., 2020), Baghdad (Al-Ameri et al., 2017) and Italy (Napolitano et al., 2013). This is lower than in the UAE (Abasaeed et al., 2009) and Sudan (Awad and Eltayeb, 2007). The difference in SMA rates may be due to differences in the demographic characteristics of the study samples, research methodology, data collection tools, and working definition of SMA. This difference may also be due to variations in pharmaceutical regulations in the respective countries. Community retail pharmacies were the primary source (71.1%) of antibiotics for students who practiced SMA. This finding implies the extensive practice of dispensing antibiotics without prescriptions. Hence, it is very important to examine the problem from the perspective of pharmacy professionals and take necessary measures, including introducing more stringent regulations on dispensing without prescription (Poyongo and Sangeda, 2020). In contrast to other studies in Iraq and Egypt (Al-Ameri et al., 2017; Helal and Abou-ElWafa, 2017), in the present study, sharing antibiotics with family and friends was minimal (8.6%).

Concerning the type of antibiotics commonly used, amoxicillin was reported as the most frequently used antibiotic among medical students (48.5%). Similar observations were also found among medical undergraduates in Ghana (Donkor et al., 2012), Northern India (Pal et al., 2016), Sri Lanka (Rathish et al., 2017) and Northwest Nigeria (Ajibola et al., 2018). The possible reasons for using amoxicillin could be its better absorption property, easy availability, inexpensiveness, effectiveness against a broad range of pathogens, and safety considerations. In this study, participants reported that diarrhea, cough, and fever were the prevailing conditions for SMA, which is in concordance with the results of other studies (Ling Oh et al., 2011; Pal et al., 2016). Most medical students cited that their academic experience, previous experience, and doctors’ prescriptions, which were advised for past illnesses, were the main sources of information about the antibiotics they used. This may increase the risk of misdiagnosis, treatment failure, and severe adverse drug reactions. Moreover, because of its previous use, the dose may also be insufficient to produce the desired effect. Similar observations, as reported by medical students, have also been observed among other medical students (Kumar et al., 2013; Mandal et al., 2020).

Among the various reasons that encouraged them to indulge in SMA practice, sufficient pharmacological knowledge or experience, followed by the problem’s urgency and saving time to visit doctors, were the most common. It is a common perception that as medical students possess sound knowledge of drugs, their pharmacological properties, and diseases, SM practice was found to be more common among them. However, in a study of medical students from West Bengal, India, saving time was the foremost reason (Banerjee and Bhadury, 2012). Another study from South India among medical professionals revealed that illness was not severe enough to consult a physician (70.5%), followed by sufficient pharmacological knowledge (45%) as the foremost reason (Kumar et al., 2013). Various common reasons for SM practices among students, as reported by other countries, were less expensive (40.5%) in Ghana (Donkor et al., 2012), and prior experience in treating similar illnesses was observed (69.6%) in Ethiopia (Eticha et al., 2014).

The current study revealed that most participants had good knowledge of antibiotics, SMA, and their implications. This points to increased expertise, while other findings show inadequate knowledge (Ocan et al., 2015) regarding SMA and its implications. Most participants were aware of the harmful effects of SMA, such as AMR and illness complications, due to delays in hospital intervention, but they still practiced SMA (Llor and Bjerrum, 2014). Although a large number of participants (96.5%) agreed that different antibiotics are needed to treat other diseases, a majority (62.8%) misunderstood that antibiotics can be used to speed up recovery from cough and cold, similar to the results obtained from Eastern Ethiopia (Rathish et al., 2017). This finding differs from that of a study conducted in Kuwait, which reported a rate of 54.4% (Awad and Aboud, 2015). The observed differences could be due to sociodemographic and setting differences. Given the widespread occurrence of upper respiratory illnesses, such an understanding can lead to an inappropriately increased use of antibiotics, adding to AMR crises.

The present study revealed a positive attitude towards taking a full course of the antibiotic regimen (69.3%) and 38.1% did not keep the antibiotics for future use. These results were lower than those of a study conducted in Eastern Ethiopia (92.1% and 87.2%, respectively). Regarding appropriate ways of getting antibiotics, the participants displayed an encouraging attitude. In this study, most respondents agreed on the need for physician consultation before purchasing antibiotics (90.4%). This finding is similar to that reported in Eastern Ethiopia (90%) (Ayele and Mamu, 2018) and higher than that reported in Saudi Arabia (76.6%) (Awad and Eltayeb, 2007; Ahmed et al., 2020). Prescription of antibiotics (55.5%) was lower than that in Eastern Ethiopia (73.1%) (Ayele and Mamu, 2018).

In this study, when asked whether they had obtained medication for their illness on their own before visiting a doctor or physician at a hospital or clinic (overall SM), 762 respondents (91.9%) answered affirmatively. There were 369 medical students (92.3%) and 393 non-medical students (91.6%) among those who responded. However, only 197 (49.1%) medical students and 254 (59.2%) non-medical students used antibiotics, indicating that non-medical students used antibiotics more than medical students. According to other research, non-medical students are more likely than medical students to self-medicate (Tesfaye et al., 2020).

SMA is more common in Tanzania’s northeast (Horumpende et al., 2018; Chuwa et al., 2021) (57%–58%) than in Karachi (Shah et al., 2014) (47%). SMA was found to be 47.8% in Southern China, 67.8% in India, and 79.5% in Sudan in other studies of SMA (Awad and Eltayeb, 2007; Sarahroodi et al., 2009; Pan et al., 2012; Shah et al., 2014; Nabi et al., 2022).

More than half of the participants (57.4%) used SM, with non-medical students having a significantly higher rate (70.1% vs. 41.1%, *p*-value <0.001). Most participants who reported self-medication did so 1–2 times a year (49.2%), with fewer reporting 3–5 times yearly (35.5%).

Pain relievers were the most commonly used self-medication drugs, followed by antibiotics. Similar to the Nepal study, the most frequently consumed drugs were antipyretics (31%), antibiotics (26.2%), analgesics (18.89%), and antihistaminics (10.1%). Similar to the Nepal study, the most commonly consumed drugs were antipyretics (31%), antibiotics (26.2%), analgesics (18.89%), and antihistaminics (10.1%). Antipyretics (31%), antibiotics (26.2%), analgesics (18.89%), and antihistaminics (10.1%) were the most commonly consumed drugs, similar to the Nepal study. Paracetamol is the most widely used drug for SM (31%), followed by azithromycin (17.6%) (Banerjee et al., 2016).

The most commonly used antibiotics among the medical and non-medical students were ampiclox (28.9% medical, 22.4% non-medical) and amoxicillin (20.8% medical, 16.1% non-medical). Ampiclox (28.9% medical, 22.4% non-medical) and amoxicillin (20.8% medical, 16.1% non-medical) were the most commonly used antibiotics among medical and non-medical students. Norfloxacin, tinidazole, ciprofloxacin, azithromycin, and cotrimoxazole are commonly used. Interestingly, in each category, medical students had a higher proportion of SM with specified antibiotics than non-medical students.

The high use of amoxicillin and ampicillin is similar to that in a study in Karachi (Shah et al., 2014), which revealed a 41.4% prevalence of amoxicillin. Medications are widely available; patients can request amoxicillin from the pharmacy. They are also readily available in any health facility (Poyongo and Sangeda, 2020; Sangeda et al., 2021; Sangeda et al., 2023a).

Due to a lack of proper knowledge, the study revealed that some students tend to use antibiotics even for diseases that do not require antibiotics or are unsure if the causes of the symptoms will be cured. For example, antibiotics are used for symptoms like cold and flu, tonsillitis, and runny nose. However, antibiotics for diarrhea or acne are among the inappropriate uses of antibiotics. Most signs or illnesses that made students self-medicate with antibiotics were diarrhea (15%), urinary tract infection (UTI) 8.8%), sore throat (8%), and tonsillitis (7%) (Shah et al., 2014).

Based on study results, most students obtain antibiotics from pharmacies, 42.9%. This shows a high distribution of pharmacies around universities and the Dar es Salaam region. Other sources included Duka la Dawa (21%), family members (2.8%), friends (2.1%), and others (0.2%). The government, pharmacy council, Tanzania Medical Devices and Drug Authority (TMDA), and other stakeholders should review their policies and even set laws and regulations to control these pharmacies, as pharmacies are leading sources where students receive antibiotics for self-medication. In contrast, many pharmacies are run by professional pharmacists who are well-trained, and they know that dispensing antibiotics without a prescription is incorrect (Poyongo and Sangeda, 2020).

The study revealed that most students (more than 35%) usually hear about self-medication from health practitioners, more than (35%) family, and friends (29%). However, most of it is through multimedia, including radio (25%), TV (35%), and the Internet (25%). Therefore, using this listed factor carefully by giving the correct information at the right time will improve students’ knowledge about antibiotics and poor self-medication practices. Observing only media (TV and radio) contributes about 60%, so organizing all media and using media better will have the best positive outcome by providing proper knowledge to society, including students. Through this rationale, the use of antibiotics can be achieved.

The mean knowledge score of the medical students was significantly higher than that of the non-medical students, indicating a better understanding of antibiotics. However, the mean attitude score of non-medical students was significantly higher than that of medical students, indicating that non-medical students had a more positive attitude towards SMA.

The knowledge, attitude, and practice of medical students regarding antibiotic use, which drives the practice of SMA, reflects a gap in the medical curricula of Tanzanian medical institutes. This pattern has also been observed in the United Arab Emirates (Jairoun et al., 2019).

Several studies in China, Ethiopia, Nepal, Pakistan, Europe, and the United States have described antibiotic-related behaviors among medical students. The SM rate ranges from 30% to 80%, and the antibiotic SM rates range from 19% to 100% (Haltiwanger et al., 2001; Pulcini et al., 2015; Scaioli et al., 2015; Ali et al., 2016; Banerjee et al., 2016; Fejza et al., 2016; Zhu et al., 2016; Hu et al., 2018; Tesfaye et al., 2020; Akande-Sholabi and Ajamu, 2021; Banda et al., 2021; Kifle et al., 2021; Nabi et al., 2022; Siraj et al., 2022).

Medical students from various universities use antibiotics in different ways. Tuyishimire et al. (2019) found that 12.1% of undergraduate university students in Rwanda self-medicate with antibiotics (Llor and Bjerrum, 2014; Tuyishimire et al., 2019). According to a study conducted at the University of Gondar in Northwest Ethiopia, medical students (34.1%) had a lower prevalence of SM than non-medical students (52.3%) (Tesfaye et al., 2020).

Medical students from different universities had different antibiotic use behaviors. Nankai University students had the highest rates of SM and stocking but the lowest rates of antibiotic demand and prophylaxis use. Students at Nankai University had the highest rates of SM and stocking but the lowest rates of antibiotic demand and prophylaxis use. Students at the university level had the highest rates of SM and stocking but the lowest rates of antibiotic demand and prophylaxis use. The highest rates of SM and stockings were found at the university level, but the lowest rates of antibiotic demand and prophylaxis use were found. Students at Nankai University had the highest rates of SM and stocking but the lowest rates of antibiotic demand and use for prophylaxis. Nankai University students had the highest rates of SM and stocking but the lowest rates of antibiotic demand and prophylaxis use. The students at Lanzhou University had the highest rates of SMA and antibiotic use. Prophylactic antibiotic use is highest in Guizhou (Hu et al., 2018).

A similar study was conducted at six universities in six regions of China to investigate university students’ antibiotic-related knowledge and behaviors (Hu et al., 2018). According to the study, 27% of people had used antibiotics to treat a self-limiting illness in the previous month.

After adjusting for medical student status, age, year of study, number of children and attitude score, it was found that being a medical student, medical students were more likely to self-medicate with antibiotics compared to non-medical students. A unit increase in age and attitude scores was also associated with SM. In a study in China, those aged between 21 and 30 years were more likely to demand antibiotics from doctors (Hu et al., 2018). Sex, marital status, having children, year of study, and knowledge score were not independent predictors of SM with antibiotics.

Prior knowledge, older age, and higher allowance are risk factors for SMA among university students in Southern China (Pan et al., 2012).

The findings of this study will help develop an intervention in training curricula in universities (Feyes et al., 2021; Varghese and Kwatra, 2023) and inform policy guidance and interventions to improve training on appropriate antibiotic use that should lead to changes in behavior. These results will have implications for training programs in medical universities and SMA practices of non-medical students as a proxy for the general population. They may help highlight areas for mass awareness campaigns regarding the inappropriate use of antibiotics. The cadres of medical students will soon become a group of healthcare workers. The impact of such knowledge has been tested during the COVID-19 pandemic, where many HCWs practiced SM with antibiotics, particularly azithromycin (Sangeda et al., 2023b; Mustafa et al., 2023).

This study had various limitations involving recall bias among the participants, as the study was based on collecting information on antibiotic usage in the 3 months to 1 year prior to the survey. The students were requested to complete the questionnaire independently; however, mutual influence could not be completely ruled out. Many students seemed to be aware that antibiotics and SMA practice are not encouraged; thus, they tend to withhold some information while practicing them, which can impact their prevalence.

## 5 Conclusion

Several studies from different parts of the world have shown that many non-medical university students use antibiotics to treat themselves. Additionally, the prevalence of SMA was slightly higher among non-medical students than medical students. This shows the importance of strengthening national educational programs and awareness campaigns, especially in medical education, to promote the right way to use antibiotics.

Medical students can be encouraged to use antibiotics smartly through seminars and lectures on how antibiotics are used and how they can lead to drug resistance. Students should be encouraged to attend such seminars and the Ministry of Health should support them. Regulatory authorities should implement appropriate antibiotic use policies involving pharmacy regulations and strategies to prevent dispensing antibiotics and other prescription-only medicines without a prescription.

The study found that pharmacies were the most common places where students received antibiotics. This shows that pharmacies must offer counseling and advice to ensure the correct use of antibiotics. SMA compared with those with moderate or low knowledge. Interestingly, despite having a higher level of knowledge about antibiotics and SM, the students were still more likely to practice. This may be due to the urgency of the problems and students’ academic knowledge. The study found that pharmacies were the most common places where students received antibiotics. This shows that pharmacies must offer counseling and advice to ensure the correct use of antibiotics.

These results can be used to create programs that warn medical students about the dangers of self-medication with antibiotics. It is very important to encourage the proper use of antibiotics and prevent the worsening of antibiotic resistance.

## Data Availability

The raw data supporting the conclusion of this article will be made available by the authors, without undue reservation.
