# Supplementary material for: Comparison of knowledge, attitude, practice and predictors of self-medication with antibiotics among medical and non-medical students in Tanzania
Source: Front Pharmacol. 2024 Jan 11;14:1301561. doi: 10.3389/fphar.2023.1301561 (PMC10808591; doi:10.3389/fphar.2023.1301561)

Supplementary Material

# Comparison of knowledge, attitude, practice and predictors of self-medication with antibiotics among medical and non-medical students in Tanzania

**Lusajo Shitindi^1^**^±^, **Omary Issa^2^**^±^, **Baraka Poyongo**^1^, **Pius Gerald Horumpende** ^3, 4, 5^, **Godeliver G Kagashe^2^, Raphael Z Sangeda^*1^**

*** Correspondence:** Corresponding Author: sangeda@gmail.com

**
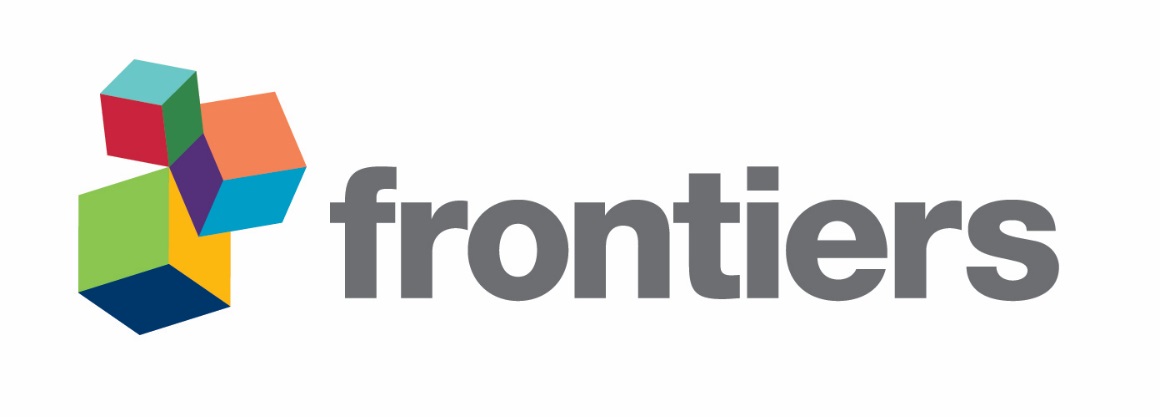
**

# Supplementary material

## INVITATION LETTER

**Dear fellow student,**

My name is **Omary Issa/** Shitindi Lusajo a fourth year student pursuing a bachelor degree of pharmacy at Muhimbili University of Health and Allied Sciences (MUHAS). I am currently conducting a survey on **Self-medication with antibiotics** for my final year research project. I kindly invite you to participate in this questionnaire survey**.** You will be required to answer questions about types and reasons for purchasing antibiotics and it’s implication with regard to the growing problem of **antimicrobial resistance**. The research will shed lights to for example the reasons for self-medication with antibiotics. **The survey (questionnaire) will take approximately 10 minutes to complete the online.**

This survey is for academic purpose, please feel free to respond to the questions because information that uniquely identify a person like his/her name or other personal information **are not asked** in this questionnaire. **All the responses will be confidential and pooled for analysis. The provided responses will be coded and no participant will be identified in any way**. Please remember, that your participation in this survey is voluntary and you can withdrawal whenever you feel uncomfortable in answering the questions.

*Since the 1940’s, antibiotics became the wonder drug and the main weapon against infectious disease caused by bacteria. However, its effectiveness can be compromised by the ability of the bacteria to become resistant to these potent molecules. Many factors may contribute to the problem. Taking medications without a prescription from a qualified physician may be one of the factors that can determine the effectiveness of drugs in killing bacteria in our body or else can contribute to antibiotic resistance.*

*As medical students we are expected to play a significant role in health care decision making and represent a major part of the community that is relatively more informed by means of the quality training we receive at the medical University. We therefore wish to and assess the level of knowledge, attitude and practice of self-medication with the antibiotics among medical students.*

*The findings from this survey on one hand will be useful to the universities to assess the current level of* ***knowledge about antibiotic use*** *among medical students and determine the need for curriculum change. On the other hand, the reasons for self-medication with antibiotics will be revealed and recommendation drew to advice the Government on strategies for the appropriate antibiotic use, appropriate behaviors changes as needed, and way for combating the ever growing problem of antimicrobial resistance and consequent failure to effectively treat bacterial infections.*

Should you have any questions or need clarifications regarding this survey, please contact **Omary Issa at +255 765362680** or by email at omaryissa20@gmail.com OR **Shitindi Lusajo at +255 755 068 702** or by email at [drhallads@gmail.com](mailto:drhallads@gmail.com)

To begin the survey, please use the electronic link below, paste the URL into the address bar of your computer or phone internet browser. A paper copy of the survey is also attached for your convenience.

<https://redcap.blood.ac.tz/sc/surveys/?s=PKYYH9WDJN>

Omary Issa/ Shitindi Lusajo, Fourth year BPharm student

Prof. Godeliver Kagashe, Research Supervisor

Dr. Raphael Sangeda, Research Co-Supervisor and Head-Department of Pharmaceutical Microbiology

School of Pharmacy, Muhimbili University of Health and Allied Sciences

## QUESTIONNAIRE

**TITLE:** ASSESSMENT OF KNOWLEDGE, ATTITUDE AND PRACTICE OF SELF-MEDICATION WITH ANTIBIOTICS AMONG MEDICAL STUDENTS AT MUHAS AND KIUT

**SECTION A: SOCIO-DEMOGRAPHIC CHARACTERISTICS**

1. Year of study ---------- Course ----------- University -------------
2. Gender ( Circle the letter corresponding to your response )
   1. Male
   2. Female
3. Age ( Circle the letter corresponding to your response )
4. 18 – 24
5. 25 – 30
6. 30+
7. Marital status (Circle the letter corresponding to your response)
8. Single
9. Married
10. Divorced
11. Widower
12. Place of residence ( put a tick to the respective answer )
13. University hostels
14. Home
15. Are you on long term medication ( put a tick to the respective answer )
16. Yes
17. No
18. If yes which kind of medication are you into? Name it -------------

**SECTION B: KNOWLEDGE ON SELF-WITH ANTIBIOTICS**

1. Have you heard of the term self-medication
2. Yes
3. No
4. Where have you heard about (Put a tick in the box corresponding to the response)

| **Sources of information** | ***Put a tick ( √ )*** |
| --- | --- |
| - - 1. Lectures |  |
| - - 1. Seminars |  |
| - - 1. Radio |  |
| - - 1. Television |  |
| - - 1. Internet |  |
| - - 1. Family or friend |  |
| - - 1. Any health practioner |  |

**SECTION C: PRACTICES OF SELF MEDICATION**

1. Have you ever practiced self-medication
   1. Yes
   2. No (***Skip to section D)***
2. If yes how is your frequency of practice
   1. Rarely (once a month)
   2. Frequently (once every 2 weeks)
   3. Very frequently (once a week)
3. Reasons for practicing self-medication ((*Put tick in the boxes corresponding to your responses)*

| - **S/N** | **REASONS** | ***Put a tick ( √ )*** |
| --- | --- | --- |
|  | Sufficient pharmacological knowledge |  |
|  | To save time |  |
|  | To avoid queue or jam at outpatient department |  |
|  | To save cost |  |
|  | Urgency of problem |  |
|  | Others (name it) |  |

13. Type of antibiotics you commonly use (*Put tick in the boxes corresponding to your responses)*

| **S/N** | **ANTIBIOTICS** | ***Put a tick ( √ )*** |
| --- | --- | --- |
|  | Amoxicillin |  |
|  | Azithromycin |  |
|  | Ampiclox |  |
|  | Ampicillin |  |
|  | Ciprofloxacin |  |
|  | Cephalexin |  |
|  | Cefuroxime |  |
|  | Co-amoxiclav (amoxicillin/clavulanic) |  |
|  | Co-trimoxazole |  |
|  | Cephalosporin |  |
|  | Tetracycline |  |
|  | Doxycycline |  |
|  | Chloramphenicol |  |
|  | Tinidazole |  |
|  | Metronidazole |  |
|  | Erythromycin |  |
|  | Septrin |  |
|  | Neomycin |  |
|  | Sulphonamide |  |
|  | Penicillin |  |
|  | Ofloxacin |  |
|  | Norfloxacin |  |
|  | Others (Mention) | |

14. Source of information about the antibiotics you took (*Put a tick in the box corresponding to your choice)*

| **S/N** | **Sources of information** | ***Put a tick ( √ )*** |
| --- | --- | --- |
|  | Academic experience |  |
|  | My own experience |  |
|  | Previous doctor’s prescription |  |
|  | Opinion of friends |  |
|  | Opinion of family members |  |
|  | Recommended by seniors |  |
|  | Antibiotic guideline |  |
|  | WHO guideline |  |
|  | Internet |  |
|  | Advertisement |  |

15. Place of obtaining the medication frequently for self medication (*Circle the number corresponding to your choice)*

1. Pharmacy store
2. Family
3. Friends
4. Supermarket

16. Symptoms or illness that made you use such antibiotics (*Put a tick in the box corresponding to your response)*

| **S/N** | **SYMPTOMS** | ***Put a tick ( √ )*** |
| --- | --- | --- |
|  | Runny nose |  |
|  | Cold and flu |  |
|  | Abscess |  |
|  | Fever |  |
|  | Sore throat |  |
|  | Acne |  |
|  | Oral ulcers |  |
|  | Wounds |  |
|  | Skin infection |  |
|  | Diarrhea and vomiting |  |
|  | Sinusitis |  |
|  | Ear infection |  |
|  | Coughs |  |
|  | Nasal congestion |  |
|  | Toothache |  |
|  | Tonsil infection |  |
|  | Aches, pain and weakness |  |
|  | Urinary tract infection |  |

17. Duration of antibiotic intake during self-medication ( *Circle the number corresponding to your choice)*

- 1. 1 – 3 days
  2. 4 – 7 days
  3. More than 7 days

| **S/N** | **Aspects of self-medication** | 1. **Yes** | 1. **No** | 1. **Don’t know** |
| --- | --- | --- | --- | --- |
|  | Different antibiotics are needed cure different diseases |  |  |  |
|  | Antibiotics are effective against bacteria |  |  |  |
|  | Antibiotics speed up the recovery from most coughs and colds |  |  |  |
|  | Antibiotics are effective against viral diseases |  |  |  |
|  | If you get side effects during a course of antibiotics treatment, you should stop taking them as soon as possible |  |  |  |
|  | If you get some kind of skin reaction when using an antibiotic, you should not use the same antibiotic again |  |  |  |
|  | The unnecessary use of antibiotics can increase resistance of bacteria to them which is the worldwide problem currently |  |  |  |
|  | The self-medication can result into harmful effects and complicate illness and sometimes results into addiction |  |  |  |
|  | Broad spectrum antibiotics are better than the narrow spectrum antibiotics |  |  |  |

18. Knowledge on aspects of self-medication (*Put a tick in the box corresponding to the response of choice*)

**SECTION D: ATTITUDE TOWARDS SELF MEDICATION**

19. What is your attitude towards self-medication? (*Put a tick corresponding to the response for every aspect of attitude towards self medication*

| **S/N** | **ATTITUDE** | **Strongly disagree** | **disagree** | **Not sure** | **agree** | **Strongly agree** |
| --- | --- | --- | --- | --- | --- | --- |
|  | Always I complete the course of treatment with the antibiotic even if I feel better |  |  |  |  |  |
|  | If I feel better after few days I sometimes stop taking my antibiotics before completing the course of treatment |  |  |  |  |  |
|  | I prefer to keep antibiotics at home or in my room in case there may be a need for them later |  |  |  |  |  |
|  | It is good to be able to get antibiotics from relatives or friends without having to see a medical doctor |  |  |  |  |  |
|  | I prefer to be able to buy antibiotics from the pharmacy without a prescription |  |  |  |  |  |
|  | I prefer to use an antibiotic of I have a cough for more than a week |  |  |  |  |  |
|  | I give the leftover antibiotics to my friends if they get sick |  |  |  |  |  |
|  | Antibiotics should be used in any case once you have a fever |  |  |  |  |  |
|  | Consultation with the physician is essential before taking any medications |  |  |  |  |  |
|  | A patient must not practice self-medication with antibiotics |  |  |  |  |  |

## QUESTIONNAIRE IN REDCap


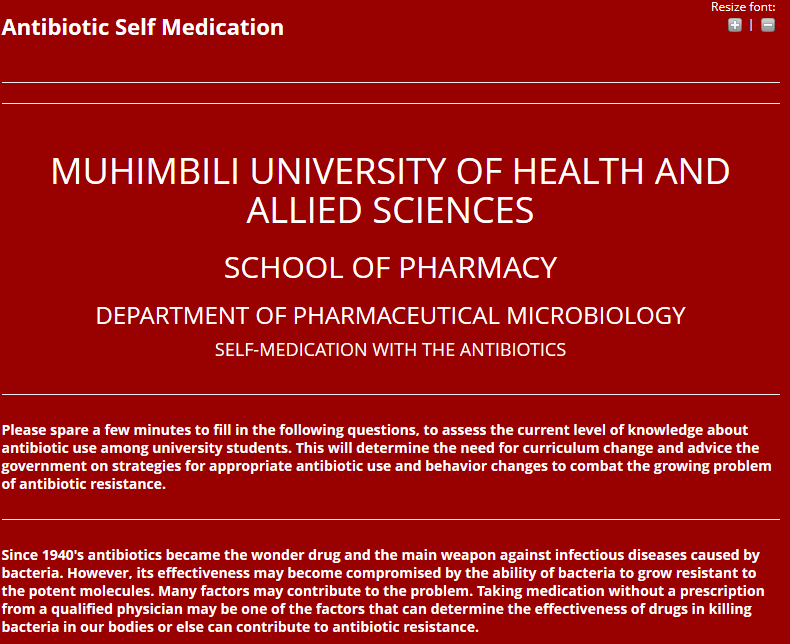


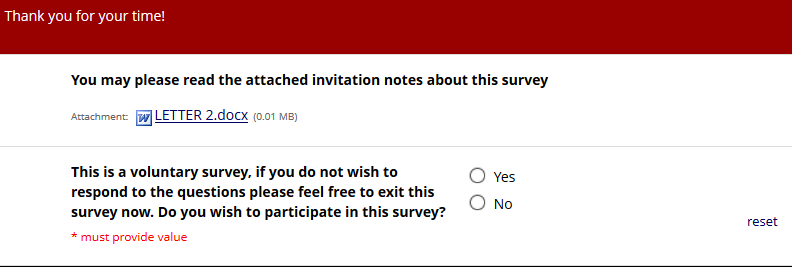


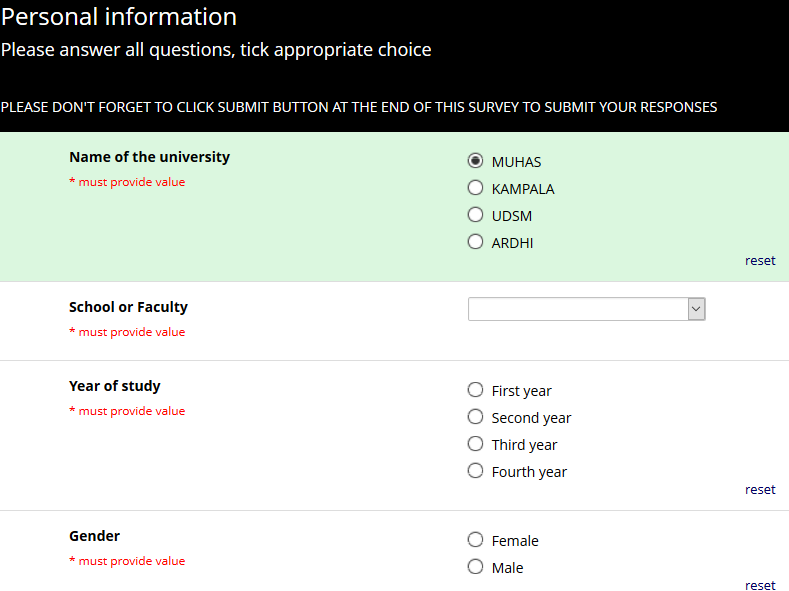


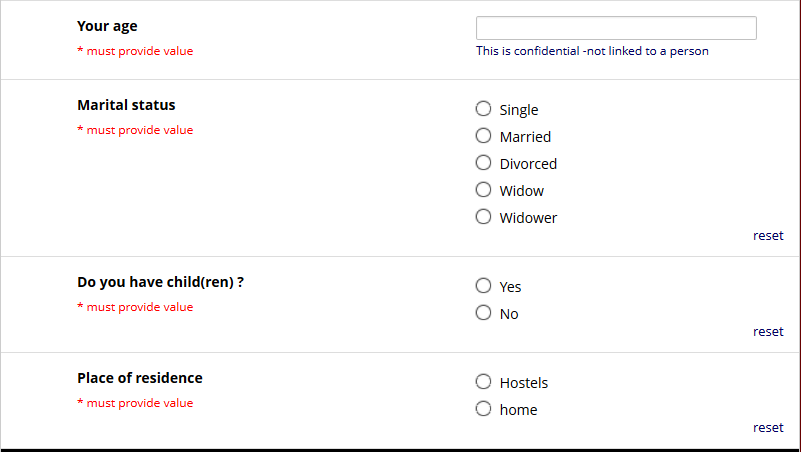


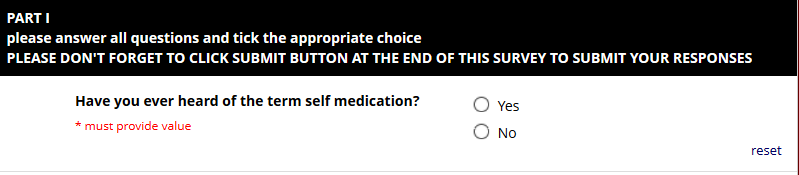


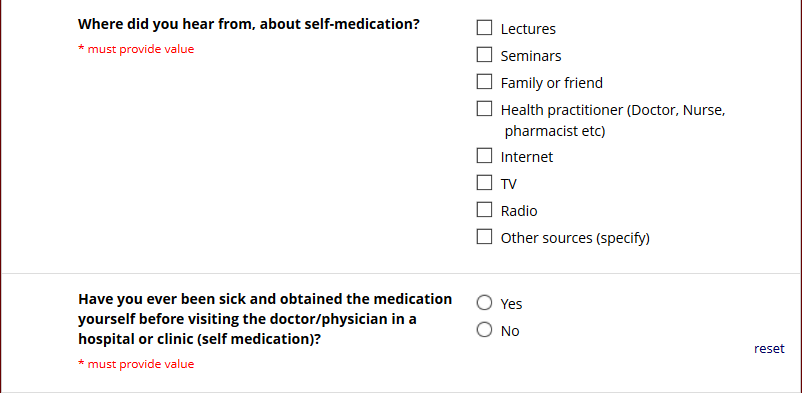


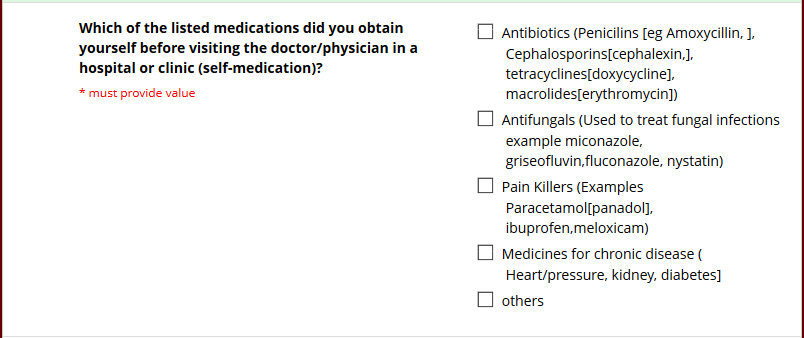


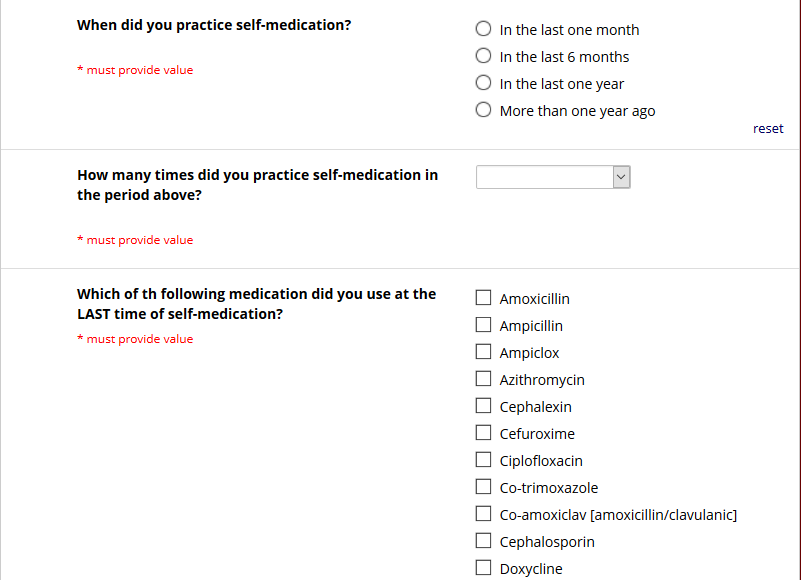


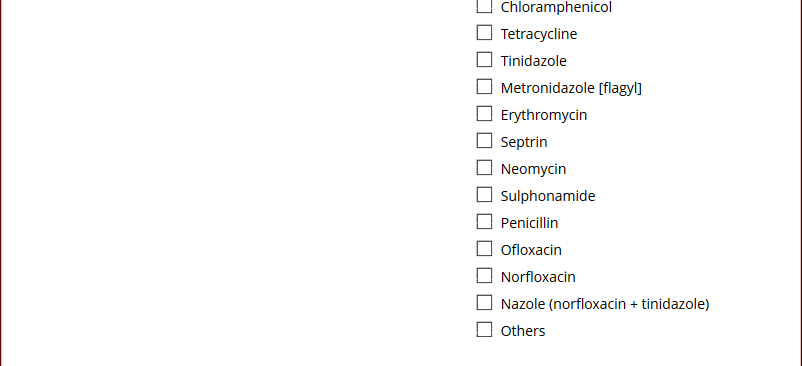


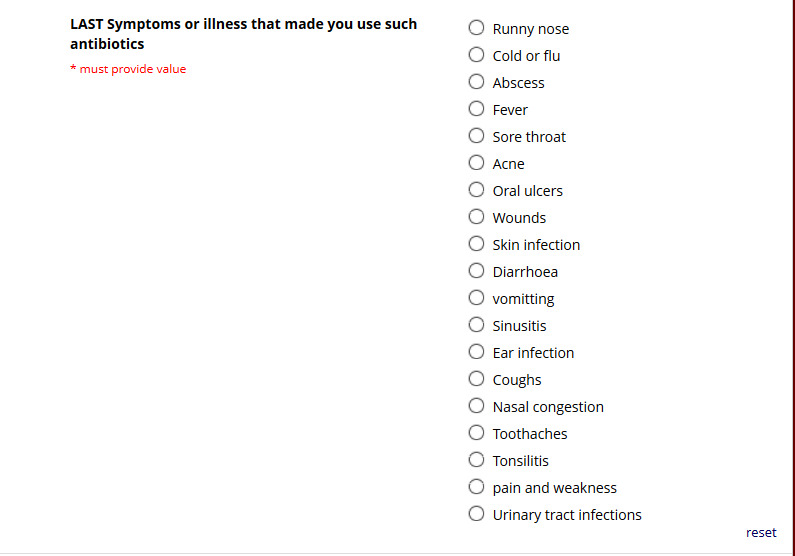


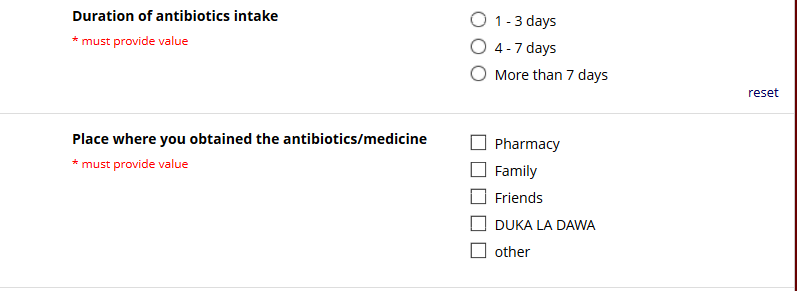


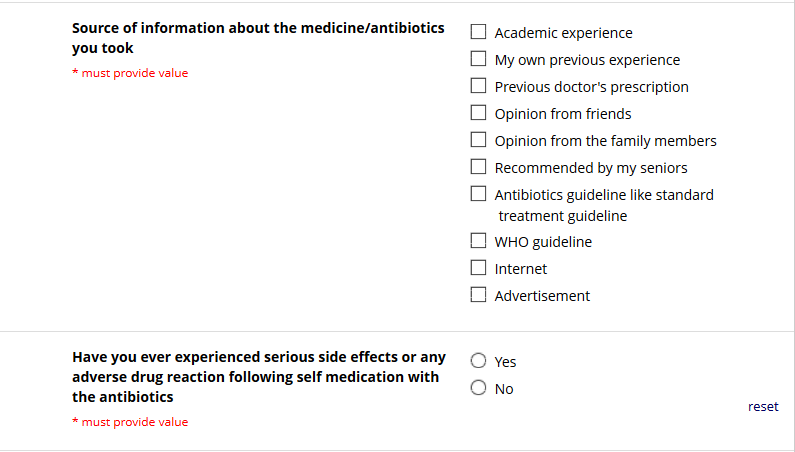


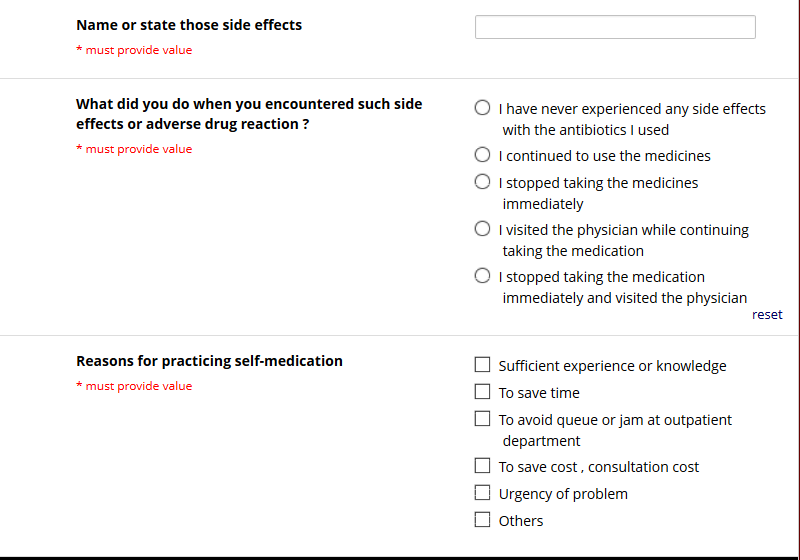


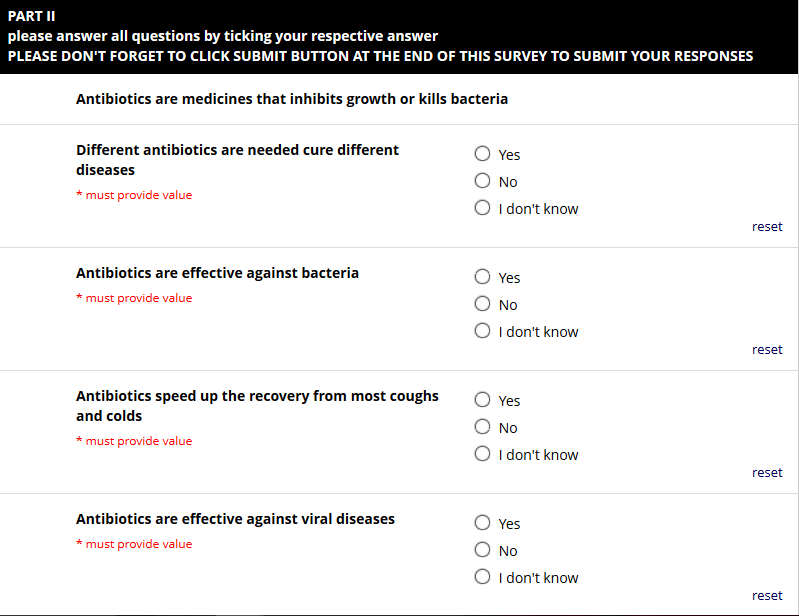


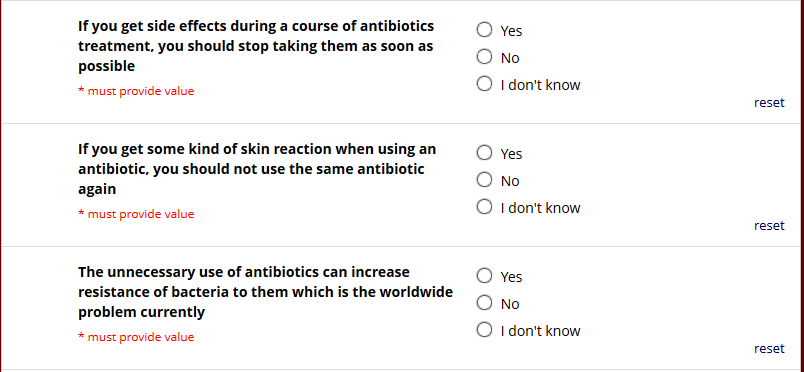


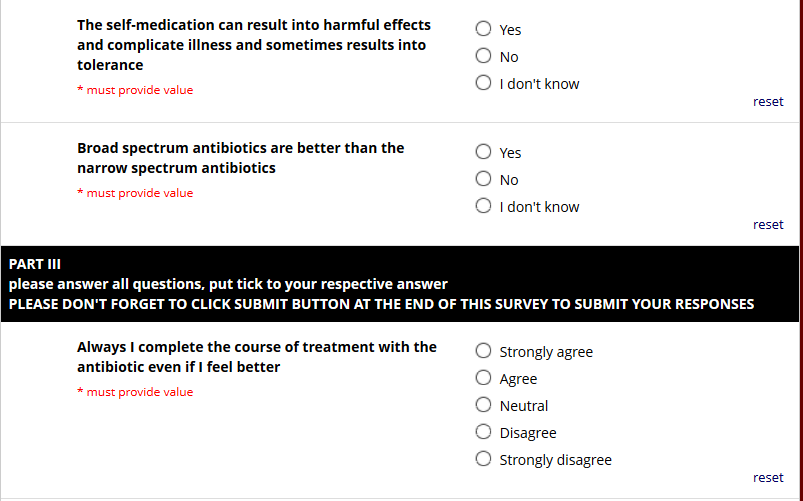


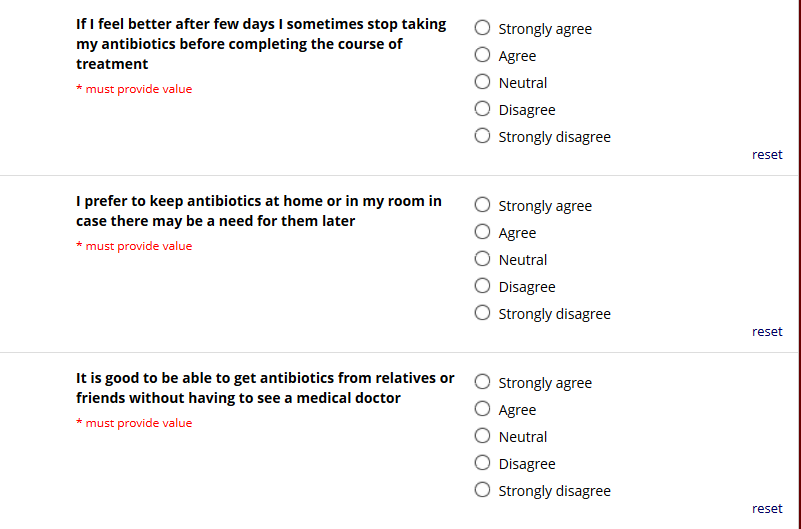


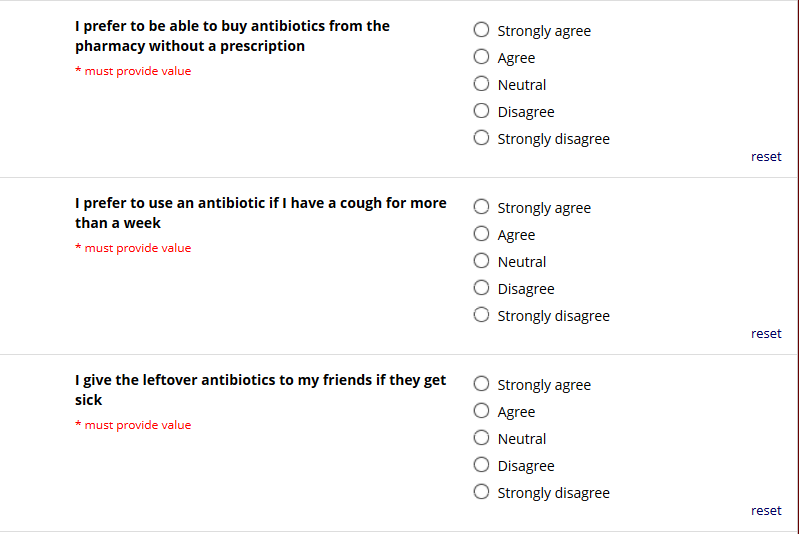

Supplement: Supplementary file 1 [file DataSheet1.docx]
